# Tumor-intrinsic CD47 signal regulates glycolysis and promotes colorectal cancer cell growth and metastasis

**DOI:** 10.7150/thno.40860

**Published:** 2020-03-04

**Authors:** Tuo Hu, Huashan Liu, Zhenxing Liang, Fengwei Wang, Chi Zhou, Xiaobin Zheng, Yunfeng Zhang, Yiwen Song, Jiancong Hu, Xiaowen He, Jian Xiao, Ryan J. King, Xianrui Wu, Ping Lan

**Affiliations:** 1 Department of Colorectal Surgery, The Sixth Affiliated Hospital, Sun Yat-sen University, Guangzhou, Guangdong, 510655, China.; 2 Guangdong Provincial Key Laboratory of Colorectal and Pelvic Floor Diseases, The Sixth Affiliated Hospital, Sun Yat-sen University, Guangzhou, Guangdong, 510655, China.; 3 Eppley Institute for Research in Cancer and Allied Diseases, University of Nebraska Medical Center, Omaha, NE 68198, USA.; 4 Guangzhou Regenerative Medicine and Health Guangdong Laboratory, Guangzhou, Guangdong, 510655, China.; 5 State Key Laboratory of Oncology in South China, Collaborative Innovation Center for Cancer Medicine, Sun Yat-sen University Cancer Center, Guangzhou, Guangdong, 510030, China.; 6 Department of Radiotherapy, The Sixth Affiliated Hospital, Sun Yat-sen University, Guangzhou, Guangdong, 510655, China.; 7 Department of Oncology, The Sixth Affiliated Hospital, Sun Yat-sen University, Guangzhou, Guangdong, 510655, China.

**Keywords:** CD47, Colorectal Cancer, ENO1, ERK, Glycolysis

## Abstract

**Rationale**: CD47 plays a vital role in the immune escape of tumor cells, but its role in regulating immune-unrelated biological processes such as proliferation and metastasis remains unclear. We seek to explore the immune-independent functions of CD47 in colorectal cancer (CRC).

**Methods**: The expression of CD47 in CRC was determined by immunohistochemistry. The biological effect of CD47 signaling on tumor cell proliferation and metastasis was evaluated *in vitro* and *in vivo*. RNA sequencing analysis was performed to identify pivotal signaling pathways modulated by CD47. The interaction between CD47 and ENO1 was verified by co-immunoprecipitation (co-IP). The effect of CD47 on glycolytic metabolites was analyzed by seahorse XF and targeted metabolomics.

**Results**: The expression of CD47 was upregulated and correlated to poor prognosis in CRC patients. Functional assays revealed that CD47 promoted CRC cell growth and metastasis *in vitro* and *in vivo*. Our mechanistic investigations demonstrated that CD47 interacted with ENO1 and protected it from ubiquitin-mediated degradation, subsequently promoting glycolytic activity and phosphorylation of ERK in CRC cells. Inhibition of ENO1 diminished CD47-mediated cell growth and migration. Clinically, the combined expression of CD47 and ENO1 provided reliable predictive biomarkers for the prognosis of CRC patients.

**Conclusions**: CD47 is overexpressed in CRC, and its expression is associated with poor prognosis. Through stabilizing ENO1, CD47 enhances the aerobic glycolysis and ERK activity in CRC cells, thereby promoting the progression of CRC. Our studies reveal an unconventional role of CD47, suggesting that targeting the CD47-ENO1 axis may provide a novel therapeutic avenue for CRC.

## Introduction

Colorectal cancer (CRC) is the third most commonly diagnosed cancer and also the third leading cause of cancer-related deaths in the United States 1. The mortality rates of CRC remain high in patients with advanced disease despite significant improvements in CRC treatment 2. Local recurrence and distant metastasis of tumors are the main causes of death in CRC patients 3. Therefore, investigating the regulatory mechanisms controlling tumor cell proliferation and metastasis as well as exploring novel key biomarkers of CRC could significantly improve the diagnosis, prevention, and treatment of CRC.

In recent years, immunotherapies have played a significant role in the efficacious treatment of multiple tumor types, resulting in remarkable improvement of cancer patient prognosis. Immune checkpoint-based therapies, including extracellular blockade of cytotoxic T lymphocyte antigen 4 (CTLA4) and programmed cell death 1 (PD1/PD-L1), exhibit tremendous clinical success in the treatment of multiple cancer types such as melanoma, leukemia, prostate cancer, and CRC 4,5. However, only a small subset of patients will respond or experience long-lasting remission to the immune checkpoint-based therapies 5,6. Therefore, it is critical to comprehensively explore the function of pertinent immune checkpoint molecules.

CD47 is an immunoglobulin-like trans-membrane protein that is ubiquitously expressed in human cells 7. The elevated expression of CD47 is found in many cancers, including gastric cancer, liver cancer, bladder cancer, lung cancer, ovarian cancer, lymphomas, and leukemia 8-11. Moreover, high level of CD47 is associated with poor prognosis in ovarian cancer, glioma, glioblastoma, and hematologic malignancies 9,12,13. Except for its role in cancer, CD47 is also reported to be involved in the pathogenesis of diet-induced obesity 14. Recent studies revealed that CD47-mediated signals play a vital role in immune-evasion of tumor cells from immune surveillance 15-17. CD47 on tumor cells binds to Signal-Regulatory Protein alpha (SIRPα) on macrophages, sequentially inhibits phagocytosis of macrophages, and attenuates the presentation of tumor antigens to T cells, thereby impairing both macrophage-mediated and cytotoxic CD8 T cell-mediated anti-tumor effects 15,16,18. Therefore, CD47 is suggested as a prominent new target in the cancer immunotherapy 9,19.

Most of the previous studies investigating CD47 focused on the immune-suppressive effects mediated by the CD47-SIRPα axis between cancer cells and macrophages. Only a few studies explored the tumor-intrinsic functions of CD47. Nonetheless, the immune-independent effects of CD47 on cancer progression were observed in some recent studies. Overexpression of CD47 had been shown to promote tumor invasion and metastasis in non-small cell lung cancer 20. High level of CD47 regulated tumor initiation, self-renewal, metastasis, and drug resistance in hepatocellular carcinoma 21,22. Moreover, the studies by Fei and Fujiwara-Tani indicated that CD47 was associated with cancer stem cell markers and thus contributed to metastasis 23, 24. Single nucleotide polymorphisms of CD47 were reported to be associated with distant metastasis and worse survival 25. On the contrary, another study demonstrated that 4N1K (the agonist ligand of CD47) could trigger selective cell death in several cancer types including CRC 26. These findings indicate that the role of CD47 signals in CRC remains not well explored. Therefore, the precise mechanism for the cellular intrinsic-function of CD47 in CRC cells needs to be clarified.

In the present study, we sought to determine the role of tumor-intrinsic CD47 signaling in the progression of CRC. We found that CD47 is significantly upregulated in CRC patients. High level of CD47 is associated with distant metastasis, recurrence, and poor prognosis. Our mechanical studies identified that CD47 protects the ubiquitin-mediated degradation of ENO1, and subsequently enhances glycolysis and activates ERK signaling, thereby promoting the proliferation and migration of CRC cells. Our data demonstrate that CD47 signaling can regulate tumor progression in a tumor-intrinsic and immune-independent manner.

## Materials and Methods

The detailed methods of the procedures are provided in Supplementary Material and Methods.

### Cell Culture

The human embryonic kidney epithelial cell line 293T (HEK293T) and human CRC cell lines (HCT8, DLD1, HCT116, SW480, HT29, HCT15, KM12, SW620, RKO) used were obtained from American type culture collection (ATCC). Cells were routinely cultured at 37°C in a 5% CO2-humidified incubator in Dulbecco's Modified Eagle's Medium (DMEM) or RPMI 1640 (Gibco, CA, USA) supplemented with 10% fetal bovine serum (Gibco, CA, USA) and 1% penicillin-streptomycin (Gibco, CA, USA). All cell lines were tested for Mycoplasma contamination every four months (last date of testing: January 25, 2019).

### Tissue Microarray and Immunohistochemistry

Paraffin-embedded CRC tissue blocks from the Sixth Affiliated Hospital of Sun Yat-sen University were used to construct tissue microarray slides. The clinical information, including TNM stage, was collected based on the pathological report. Survival information was retrieved from telephone follow-ups or medical records. Our study was approved by the ethics committee of the Sixth Affiliated Hospital of Sun Yat-sen University. Immunohistochemistry (IHC) staining was performed to detect the expression levels of indicated proteins. The detailed procedures of IHC are provided in Supplementary Material and Methods.

### Plasmid Construction and Cell Transduction

The specific primers used for plasmid construction are listed in Supplementary Table 4. The detailed procedures for plasmid construction and cell transduction are provided in the Supplementary Material and Methods.

### *In Vivo* Experimentation

To conduct the *in vivo* experiments, four-week-old male athymic nude mice were purchased and maintained in pathogen-free conditions at the Experimental Animal Center in Sun Yat-sen University. All animal experiments were conducted in accordance with the approval of the Institutional Animal Care and Use Committee of Sun Yat-sen University. The detailed information of the animal experiments is provided in the Supplementary Material and Methods.

### High-Throughput Sequencing and Bioinformatic Analysis

Total RNA from CD47-overexpressed DLD1 and HCT8 cells was extracted by RNeasy Mini Kit (Qiagen, Germany) and subsequently tested by an Agilent 2100 Bioanalyzer for quality control. The RNA samples were processed for sequencing on the BGISEQ-500 platform by Beijing Genomics Institute using a previously described detailed procedure for RNA sequencing 27. Pathway analysis utilizing the Gene Ontology (GO) and Kyoto Encyclopedia of Genes and Genomes (KEGG) was conducted by R to identify the biological pathways regulated by overexpression of CD47. P value less than 0.05 was regarded as statistically significant.

### Co-immunoprecipitation (co-IP), Immunopurification and Mass Spectrometry

HEK293T cells transfected with Flag-CD47 were immunoprecipitated with anti-Flag magnetic beads and processed for proteomic data analysis by liquid chromatography tandem mass spectrometry (LC-MS/MS). For co-IP assays, cell lysates were immunoprecipitated with the indicated magnetic beads and co-IP samples were then processed and visualized by immunoblotting assays. The detailed procedures of co-IP are provided in Supplementary Material and Methods.

### Statistical Analysis

All data are shown as mean ± standard deviation (SD) from at least three independent experiments. SPSS 22.0 was used to perform the statistical analysis. The detailed information of statistical analysis is provided in the Supplementary Material and Methods.

## Results

### CD47 increases proliferation and metastasis of CRC cells

We checked the protein and mRNA levels of CD47 in one immortalized human non-tumorigenic intestinal epithelial cell line (NCM460) and six common used CRC cell lines. The mRNA expression of CD47 was significantly increased in CRC cells compared to non-tumorigenic intestinal epithelial cells (Figure S1A). Among cancer cells, HCT116 and SW480 had a relatively higher level of CD47, while HCT8 and DLD1 cells exhibited a lower level of CD47 (Figure S1A-B). Consequently, HCT116 and SW480 cells were used to construct CD47 knockdown cell lines, while HCT8 and DLD1 cells were chosen to construct CD47 overexpression cell lines. The efficiency of CD47 overexpression and knockdown was validated by qRT-PCR and western blot (Figure S1C-F). In HCT8 and DLD1 cells containing low expression of CD47, overexpression of CD47 significantly promoted cell migration, invasion, and proliferation compared to control cells (Figure S2A-D). In contrast, short hairpin RNA (shRNA) mediated-knockdown of CD47 dramatically impaired the capabilities of proliferation, migration, and invasion in HCT116 and SW480 cells (Figure S2E-H).

**Figure 1:**
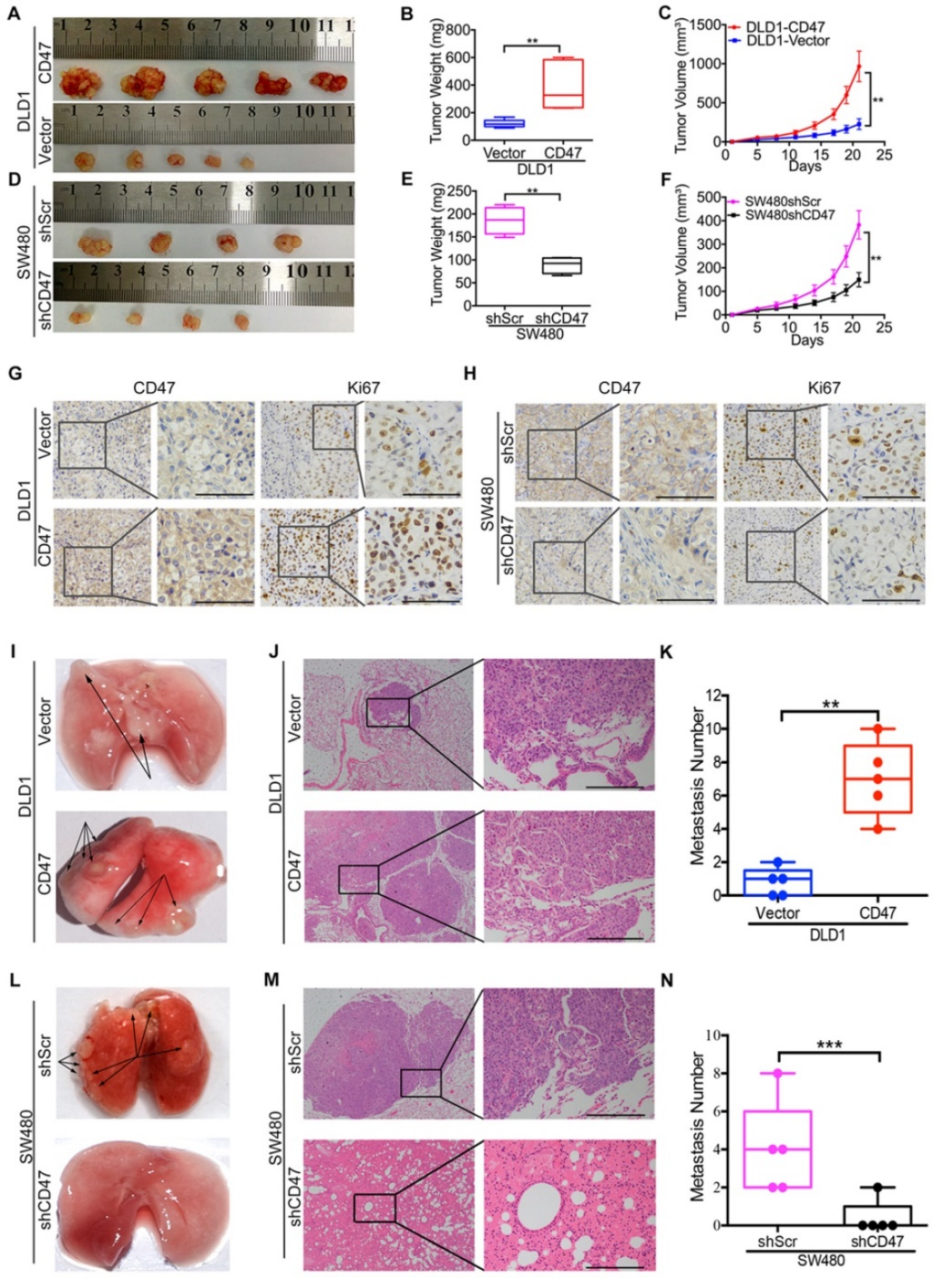
** CD47 enhances CRC cell proliferation and metastasis *in vivo*.** (A-H). CD47 promotes growth of CRC cells *in vivo.* DLD1-Vector, DLD1-CD47, SW480-shScramble, and SW480-shCD47 CRC cells were subcutaneously injected into nude mice (n = 5 and 4 respectively). Images of tumors (A, D), statistics of tumor weights (B, E), tumor volumes (C, F), and representative IHC staining are shown (G, H). (I-N). CD47 increases tumor metastasis *in vivo*. DLD1-Vector, DLD1-CD47, SW480-shScramble, and SW480-shCD47 cells were injected into tail veins of nude mice (n = 5). Representative images (I, L), HE staining (J, M), and statistics of lung metastatic tumors are depicted (K, N). Data are the means ± SD, *p<0.05, **p<0.01, ***p<0.001.

To explore the CD47 function independent of CD47-SIRPα axis *in vivo*, clodrolate liposomes were used to delete macrophages in nude mice (Figure S3A). Macrophage depletion efficiency was confirmed by the significant decrease of the percentage of F4/80^+^/CD11b^+^ cells in the spleen (Figure S3B-C). All of the *in vivo* experiments were conducted in nude mice with macrophages deletion in this study. The subcutaneous xenograft model was used to investigate the effects of CD47 on tumor cell growth of CRC *in vivo*. Consistent with the* in vitro* results, overexpression of CD47 increased tumor growth in DLD1 cells compared to control cells (Figure 1A-C), while knockdown of CD47 inhibited tumor cell growth in SW480 cells (Figure 1D-F). In consistent with the differences of tumor growth rate, the positive ratio of Ki67, a cell division marker, was increased in CD47-overexpressing DLD1 tumors but decreased in CD47-knockdown SW480 tumors compared to their corresponding control groups (Figure 1G-H).

We then used pulmonary metastasis model to explore the role of CD47 in CRC cell metastasis *in vivo*. A greater number of metastatic nodules were observed in the DLD1 CD47-overexpressing group in comparison with the control group (Figure 1I-K, Figure S3D). In contrast, less metastatic nodules were found in the SW480 CD47-knockdown group compared to the control group (Figure 1L-N, Figure S3E). Together, our data clearly showed that tumor-intrinsic CD47 could promote CRC cell proliferation and metastasis *in vitro* and *in vivo*.

### CD47 enhances aerobic glycolysis and ERK activation by upregulating ENO1

**Figure 2:**
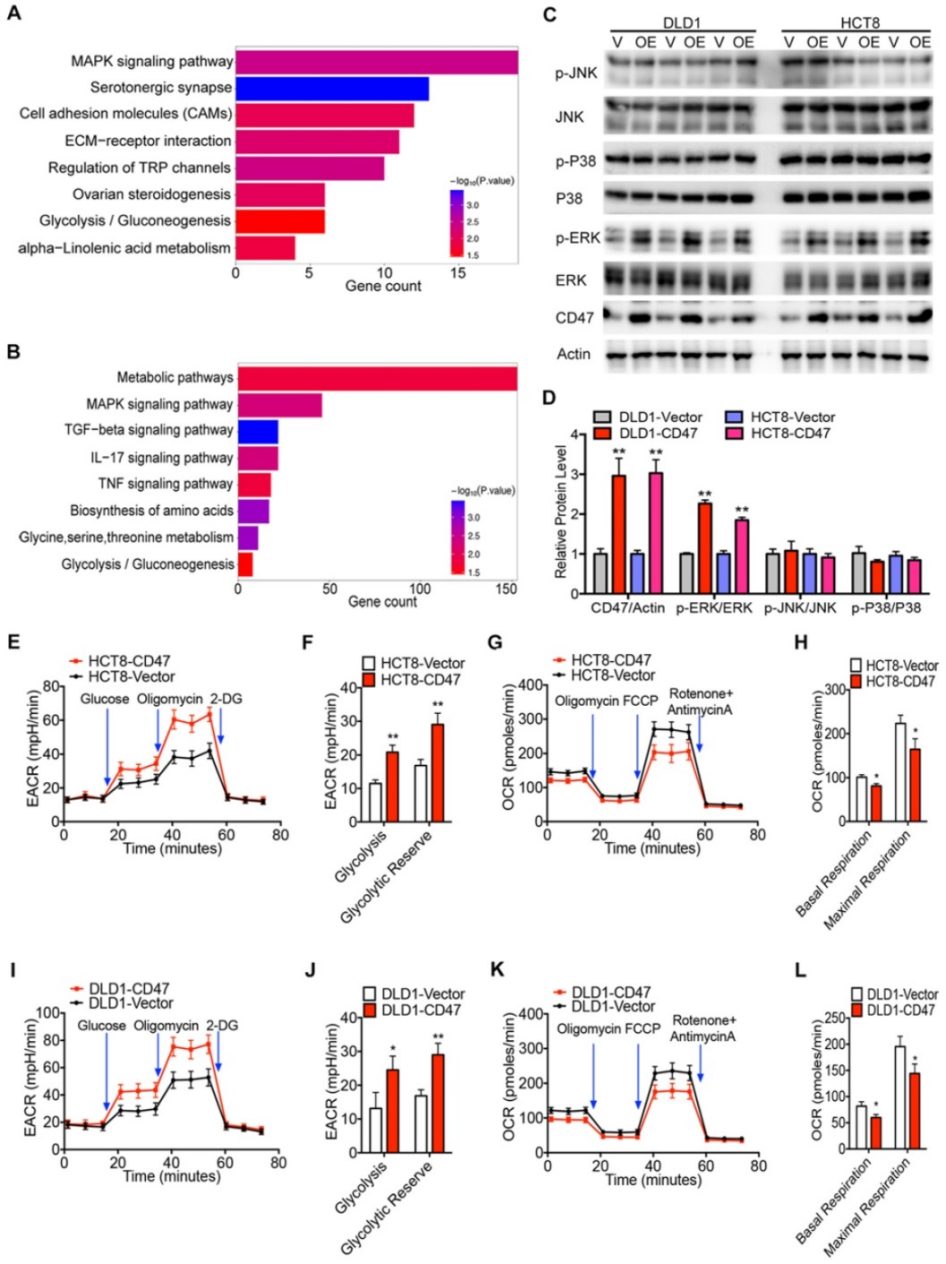
** CD47 increases ERK phosphorylation and glycolysis.** (A-B). KEEG pathway significance of genes regulated by CD47 overexpression in DLD1 (A) and HCT8 (B) cells. Enriched pathways are listed by their gene count and p-value. (C-D). Immunoblotting and relative protein levels of JNK (Thr183/Tyr185), p38 (Thr180/Tyr182), and ERK (Thr202/Tyr204) phosphorylation in DLD1 and HCT8 cells stably overexpressing CD47, β-actin was used as the internal control, OE overexpression. (E-H). Extracellular acid ratio (ECAR) and Oxygen consumption rate (OCR) of HCT8-Vector and HCT8-CD47 cells were measured using Seahorse XF. 2-DG, 2-deoxyglucose. Carbonyl cyanide 4-(trifluoromethoxy) phenylhydrazone, FCCP. (I-L). ECAR and OCR were tested in DLD1-Vector and DLD1-CD47 cells. Data are the means ± SD, *p<0.05, **p<0.01, ***p<0.001.

RNA-seq analysis was performed to identify the related biological signaling pathways which might be involved in CD47-mediated CRC cell proliferation and metastasis. Differentially expressed genes in control and CD47-overexpressed HCT8 and DLD1 cells were illustrated in the scatter-plot with FDR<0.001 and fold change >2 (Figure S4A-B). GO analysis indicated that CD47 regulated a variety of biological process in CRC cells, specifically, processes associated with cell proliferation and migration (Figure S4C-D). Moreover, KEGG pathway analysis showed that MAPK signaling pathway and glycolysis/gluconeogenesis pathway were remarkably enriched in CD47 overexpression cells (Figure 2A-B).

To confirm these findings, we examined the effect of CD47 overexpression on MAPK signaling pathway by immunoblotting. Overexpression of CD47 significantly increased the phosphorylation level of ERK (Thr202/Tyr204), but not JNK (Thr183/Tyr185) and p38 (Thr180/Tyr182) in HCT8 and DLD1 cells, while total amounts of ERK, JNK, and p38 protein levels remained unchanged (Figure 2C-D). We also determined whether CD47 overexpression influenced glycolytic metabolism in CRC cells by measuring the extracellular acidification rate (ECAR) and oxygen consumption rate (OCR). Indeed, overexpression of CD47 significantly increased ECAR levels in HCT8 and DLD1 cells compared to control cells (Figure 2E-F, Figure 2I-J). Meanwhile, decreased OCR levels were observed in HCT8 and DLD1 CD47-overexpression cells compared to vector cells (Figure 2G-H, Figure 2K-L). Moreover, CD47 overexpression substantially increased the level of several glycolytic metabolites including phosphoenol pyruvate (PEP), pyruvate, and lactate in DLD1 cells (Figure S4E). On the contrary, CD47 knockdown significantly decreased the level of PEP, pyruvate, and lactate in SW480 cells (Figure S4F). These data indicate that CD47-overexpression increases aerobic glycolysis and ERK activation in CRC cells.

**Figure 3:**
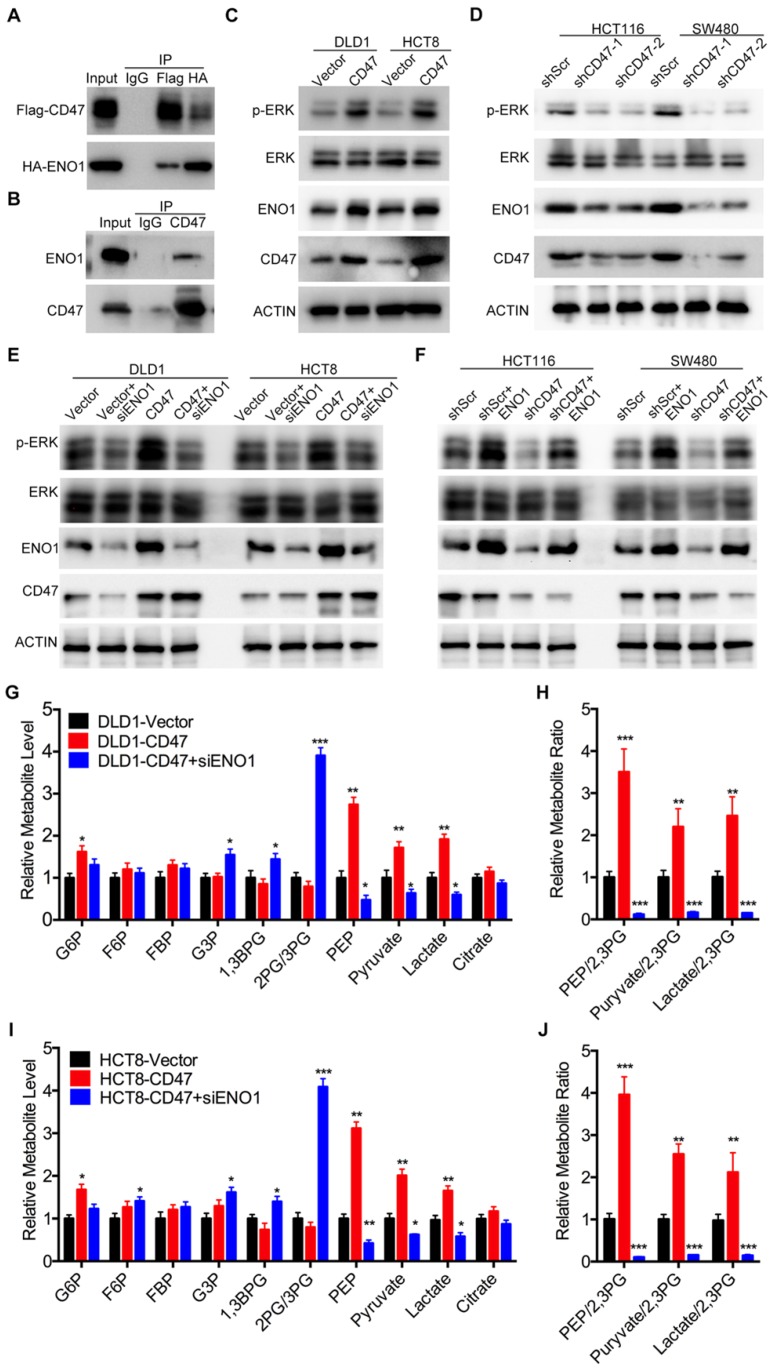
** CD47 enhances aerobic glycolysis and activates ERK pathway by upregulating ENO1.** (A-B). CD47 interacts with ENO1. HEK293T cells were transfected with Flag-CD47 and HA-ENO1 plasmids, cells lysates were immunoprecipitated with anti-Flag/HA magnetic beads and then immunoblotted with the indicated antibodies (A). SW480 cells lysates were immunoprecipitated with anti-CD47 antibody and immunoblotted with CD47 and ENO1 antibodies (B). (C-D). Immunoblotting of ENO1 and ERK (Thr202/Tyr204) phosphorylation in DLD1 and HCT8 cells stably overexpressing CD47 (C) or in stable CD47 knockdown HCT116 and SW480 cells (D).(E-F). Rescue assays for immunoblotting of ERK (Thr202/Tyr204) phosphorylation after ENO1 silencing in DLD1 and HCT8 cells stably overexpressing CD47 (E) or after transiently expressing ENO1 in stable CD47-knockdown HCT116 and SW480 cells (F) β-actin was used as the internal control. (G-H). Relative metabolite levels (C) and ratios (D) in glycolysis and TCA cycle from DLD1-Vector, DLD1-CD47 and DLD1-CD47+siENO1 cells. (I-J). Relative metabolite levels (E) and ratios (F) in glycolysis and TCA cycle from HCT8-Vector, HCT8-CD47 and HCT8-CD47+siENO1 cells. Data are the means ± SD, *p<0.05, **p<0.01.

Since CD47 is a transmembrane protein, we hypothesized that CD47 might regulate the MAPK/ERK and glycolysis pathways via some intermediate molecules. To verify our hypothesis, we determined CD47-interacting proteins by using co-IP and subsequent mass spectrometry analysis. Six potential CD47-interacting proteins were identified from three independent experiments (Figure S5A). Among these CD47-interacting proteins, ENO1, a glycolytic enzyme, has been reported to regulate proliferation and metastasis of CRC 28,29. We suspected ENO1 might be a downstream target of CD47 and mediate some functions of CD47. Immunofluorescence assays illustrated that CD47 co-localized with ENO1 in HCT116 and SW480 cells (Figure S5C). The interaction between CD47 and ENO1was further confirmed by co-IP assay in HEK293 cells as well as SW480 cancer cells (Figure 3A-B). Interestingly, the protein level of ENO1 was significantly increased in CD47-overexpressed HCT8 and DLD1 cells, but decreased in CD47-knockdown HCT116 and SW480 cells compared to their corresponding cells (Figure 3C-D).

ENO1 is a glycolytic enzyme which converts phosphoglycerate (2-PG) to phosphoenolpyruvate (PEP) 30. Previous studies showed that ENO1 was able to increase the phosphorylation level of AMPK, AKT and ERK 29,31,32. As anticipated, knockdown of ENO1 decreased the phosphorylated ERK (Thr202/Tyr204) levels in HCT116 and SW480 cells (Figure S5B). Therefore, we speculated that ENO1 might be involved in CD47-mediated ERK phosphorylation in CRC cells. As expected, the increased ERK phosphorylation in CD47-overexpressed cells was diminished by ENO1 silencing (Figure 3E). Moreover, transiently expressing ENO1 rescued the decrease of ERK phosphorylation in CD47-knockdown HCT116 and SW480 cells (Figure 3F). We further investigated whether ENO1 is also involved in CD47-mediated enhancement of glycolysis. CD47 overexpression significantly increased the level of glucose 6-phosphate (G6P), phosphoenol pyruvate (PEP), pyruvate, and lactate in DLD1 cells. ENO1 knockdown blocked CD47-induced increase of PEP, pyruvate, and lactate in CD47-overexpressed DLD1 cells (Figure 3G-H). The similar results were also found in HCT8 cells (Figure 3I-J). Taken together, our findings indicate that ENO1 is a target molecule of CD47 and mediates CD47-induced ERK phosphorylation and glycolysis elevation.

### CD47 prevents the degradation of ENO1 through inhibiting FBXW7

**Figure 4:**
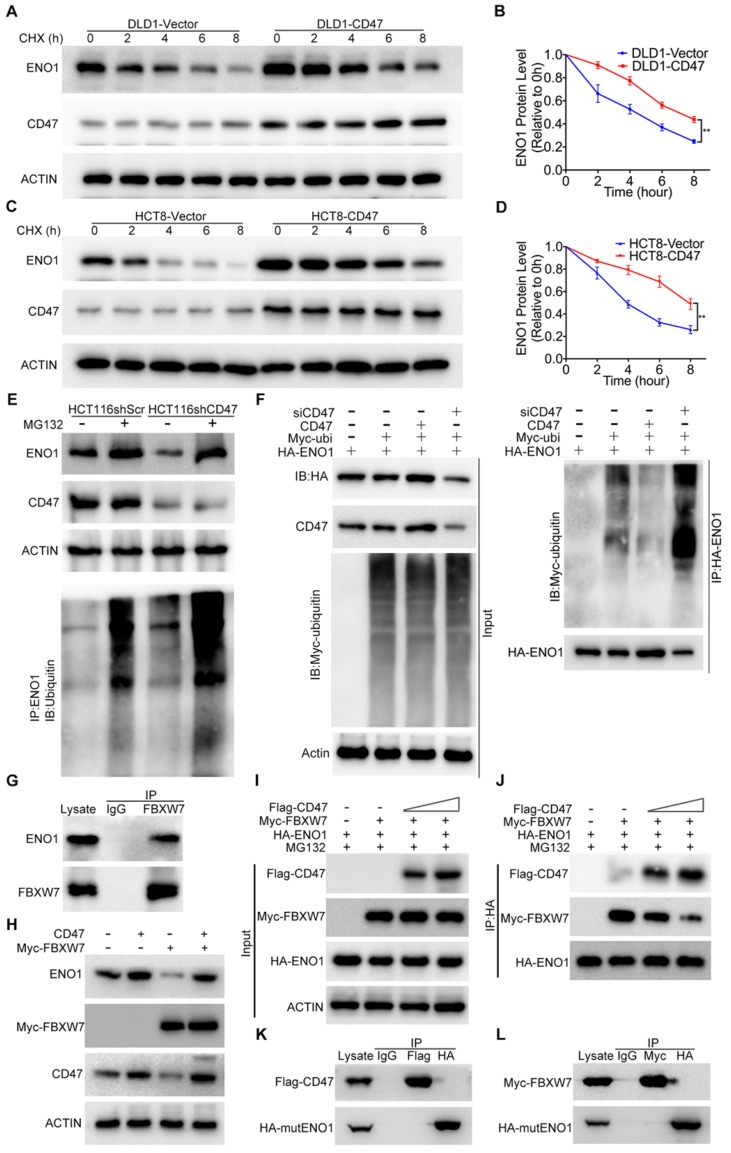
** CD47 inhibits FBXW7-mediated ubiquitylation and degradation of ENO1.** (A-B). Immunoblotting and quantitative analysis for ENO1 protein levels in CD47-overexpressed DLD1 cells treated with cycloheximide (CHX, 50 µg/mL). (C-D). Immunoblotting and quantitative analysis for ENO1 protein levels in CD47-overexpressed HCT8 cells treated with CHX (50 µg/mL). Data are the means ± SD. (E). ENO1 protein level and endogenous ENO1 ubiquitination level in control and CD47 knockdown HCT116 cells treated with MG132 (25 µM) for 12 h. (F). HT29 cells were transfected with indicated plasmids and siRNA. MG132 (25μM) was added 6h before harvest. Cell lysates were immunoprecipitated with anti-HA magnetic beads and detected by anti-ubiquitin antibody. The input levels of CD47, HA-ENO1, ubiquitin and actin are shown. (G). SW480 cells lysates were immunoprecipitated with anti-FBXW7 antibody and immunoblotted with FBXW7 and ENO1 antibodies. (H). Immunoblotting for ENO1 protein levels in DLD1-Vector and DLD1-CD47 cells transiently expressing Myc-FBXW7. (I-J). HEK293T cells were transfected with the indicated plasmids and MG132 (25μM) was added simultaneously during transfection. Cell lysates were immunoprecipitated with anti-HA magnetic beads and then immunoblotted by the indicated antibodies (J). The input levels of Flag-CD47, Myc-FBXW7 and HA-ENO1 are shown (I). (K). HEK293T cells were transfected with CD47-Flag and mutant ENO1-HA plasmids. Cell lysates were immunoprecipitated with anti-Flag/HA magnetic beads and then immunoblotted by the indicated antibodies. (L). HEK293T cells were transfected with FBXW7-Myc and mutant ENO1-HA plasmids. MG132 (25 µM) was added 6h before harvest. Cell lysates were immunoprecipitated with anti-Myc/HA magnetic beads and then immunoblotted by the indicated antibodies.

Overexpression of CD47 increased the protein level of ENO1 in DLD1 and HCT8 cells (Figure 3C). However, the mRNA levels of ENO1 remained unaltered in CD47-overexpressed cells compared to control cells (Figure S5D), indicating ENO1 upregulation may be through a post-transcriptional mechanism by CD47. ENO1 protein level was significantly reduced after treatment with the protein synthesis inhibitor cycloheximide (CHX) in control DLD1 and HCT8 cells, but overexpression of CD47 partially rescued CHX-induced reduction of ENO1 (Figure 4A-D). In contrast, accelerated degradation of ENO1 was observed in CD47-knockdown HCT116 and SW480 cells (Figure S6A-D). The degradation of proteins mainly depends on the proteasome system 33. Interestingly, proteasome inhibitor MG132 blocked CD47 knockdown-induced turnover of ENO1 in HCT116 and SW480 cells. Moreover, the endogenous ENO1 ubiquitylation level was increased in CD47 knockdown cells compared to control cells both regardless of MG132 (Figure 4E, Figure S6G). On the contrary, CD47-overexpression inhibited the turnover of ENO1 in HCT8 and DLD1 cells (Figure S6E-F). These results suggest that CD47 may increase the stability of ENO1 by inhibiting the proteasome-mediated degradation of ENO1 in CRC cells. We then investigated whether CD47 regulated the ubiquitylation level of ENO1. As anticipated, the ubiquitylation level of ENO1 was distinctly decreased in CD47-overexpressed HT29 colon cancer cells but increased in CD47-knockdown HT29 cells (Figure 4F).

A recent study showed that FBXW7, an E3 ubiquitin ligase, could interact with ENO1 and mediate its ubiquitylation 28. We confirmed the interaction between FBXW7 and ENO1 using co-IP assay (Figure 4G and Figure S6H). Moreover, the endogenous ENO1 ubiquitylation level was decreased in FBXW7 knockdown cells but increased in FBXW7 overexpression cells when compared to their corresponding control cells (Figure S6I-J). Consistent with the previous study, overexpression of FBXW7 decreased ENO1 in DLD1 cells, but overexpression of CD47 protected ENO1 from FBXW7-mediated degradation (Figure 4H). Therefore, we speculated that CD47 stabilized ENO1 by inhibiting its ubiquitylation mediated by FBXW7. However, co-IP results suggested that there was no binding between CD47 and FBXW7 (Figure S6K). Furthermore, the expression of FBXW7 remained unaltered following CD47 overexpression in HCT8 and DLD1 cells (Figure S6L). Therefore, we hypothesized that the CD47 might competitively prevent ENO1 binding to FBXW7. To test this hypothesis, HEK293T cells were transfected with the same concentration of HA-ENO1 and Myc-FBXW7 but increasing concentrations of Flag-CD47 plasmids for subsequent co-IP assays. With the increasing expression of CD47 (Flag), less FBXW7 (Myc) was pulled down by ENO1 (HA) (Figure 4I-J), suggesting that CD47 impaired the interaction of FBXW7 and ENO1. ENO1 contains a typical FBXW7-targeted motif known as the CDC4 phospho-degron (CPD), X-pT/pS-P-(P)-X-X-pS/pT/E/D (X represents any amino acid, Asparte (D), Glutamate (E), Proline (P), and pT/S means phosphorylated Threonine/Serine) 34,35. We generated a mutant construct by mutating the CPD motif (S263A/S268A) of HA-ENO1 (Figure S6M-N). Both CD47 and FBXW7 could bind to wild type ENO1 (Figure 3A-B, Figure 4G), but neither CD47 nor FBXW7 was able to bind with the mutant HA-ENO1 (S263A/S268A) (Figure 4K-L), indicating that CD47 competes with FBXW7 for the CPD motif of ENO1. Taken together, our findings demonstrate that CD47 impedes the interaction between FBXW7 and ENO1, and thereby inhibits FBXW7-mediated ubiquitylation and degradation of ENO1.

### ENO1 is essential for CD47-mediated enhancement of proliferation and metastasis in CRC cells

**Figure 5:**
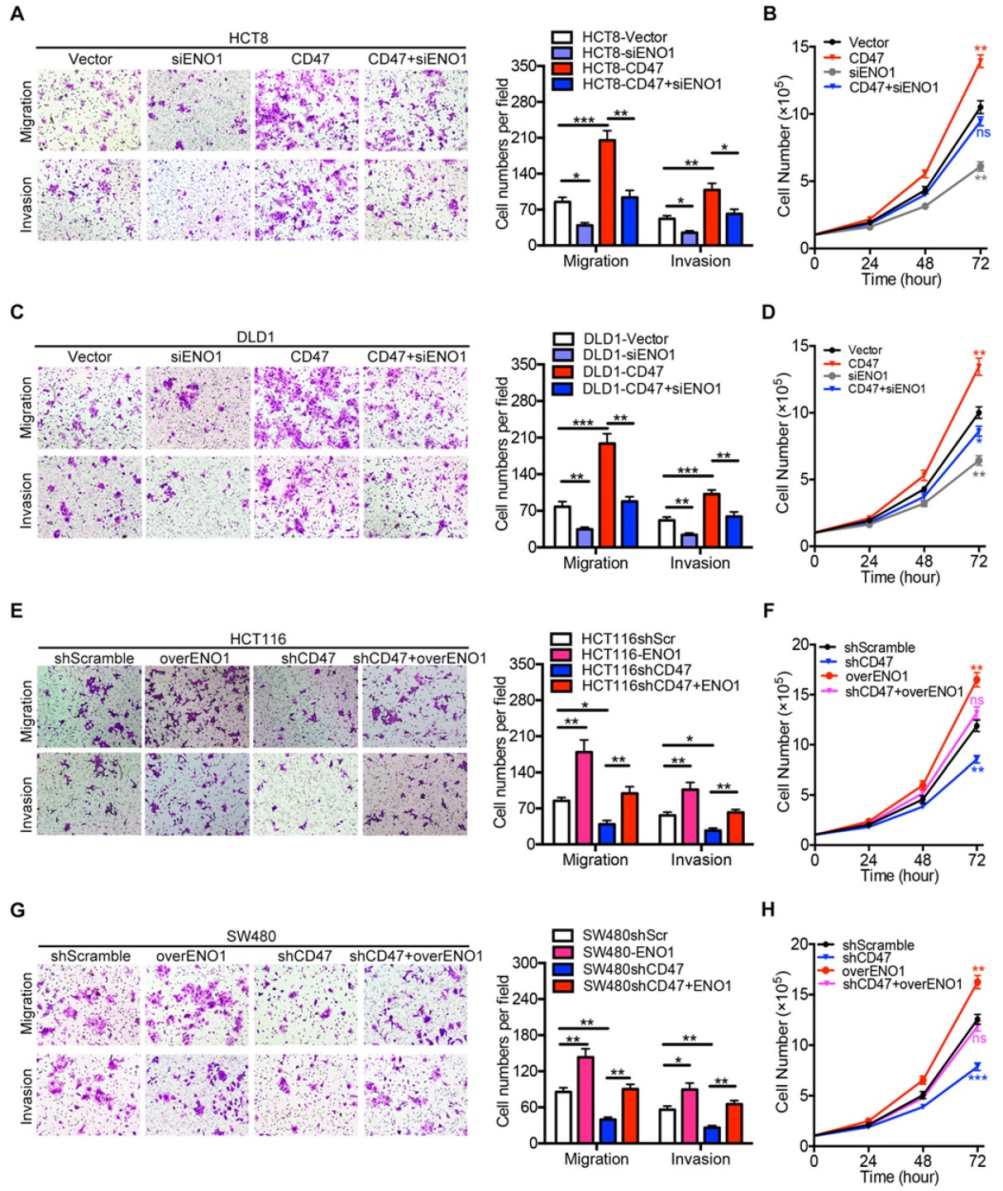
** Rescue assays of CRC cell proliferation, migration, and invasion *in vitro*.** (A-D). Transwell migration /invasion assays and cell proliferation assays were performed after ENO1 silencing in control and CD47-overexpressed DLD1/HCT8 cells. (E-G). Transwell migration/invasion assays and cell proliferation assays were performed after transiently expressing ENO1 in control and CD47-knockdown HCT116/SW480 cells. Data are means ± SD, *p<0.05, **p<0.01, ***p<0.001.

We further investigated the role of ENO1 in CD47 regulating proliferation and metastasis of CRC cells. ENO1 silencing inhibited the effects of CD47 overexpression on proliferation, migration, and invasion in HCT8 and DLD1 cells (Figure 5A-D). Furthermore, ENO1 overexpression rescued the inhibition of proliferation, migration, and invasion mediated by CD47 knockdown in HCT116 and SW480 cells (Figure 5E-H).

**Figure 6:**
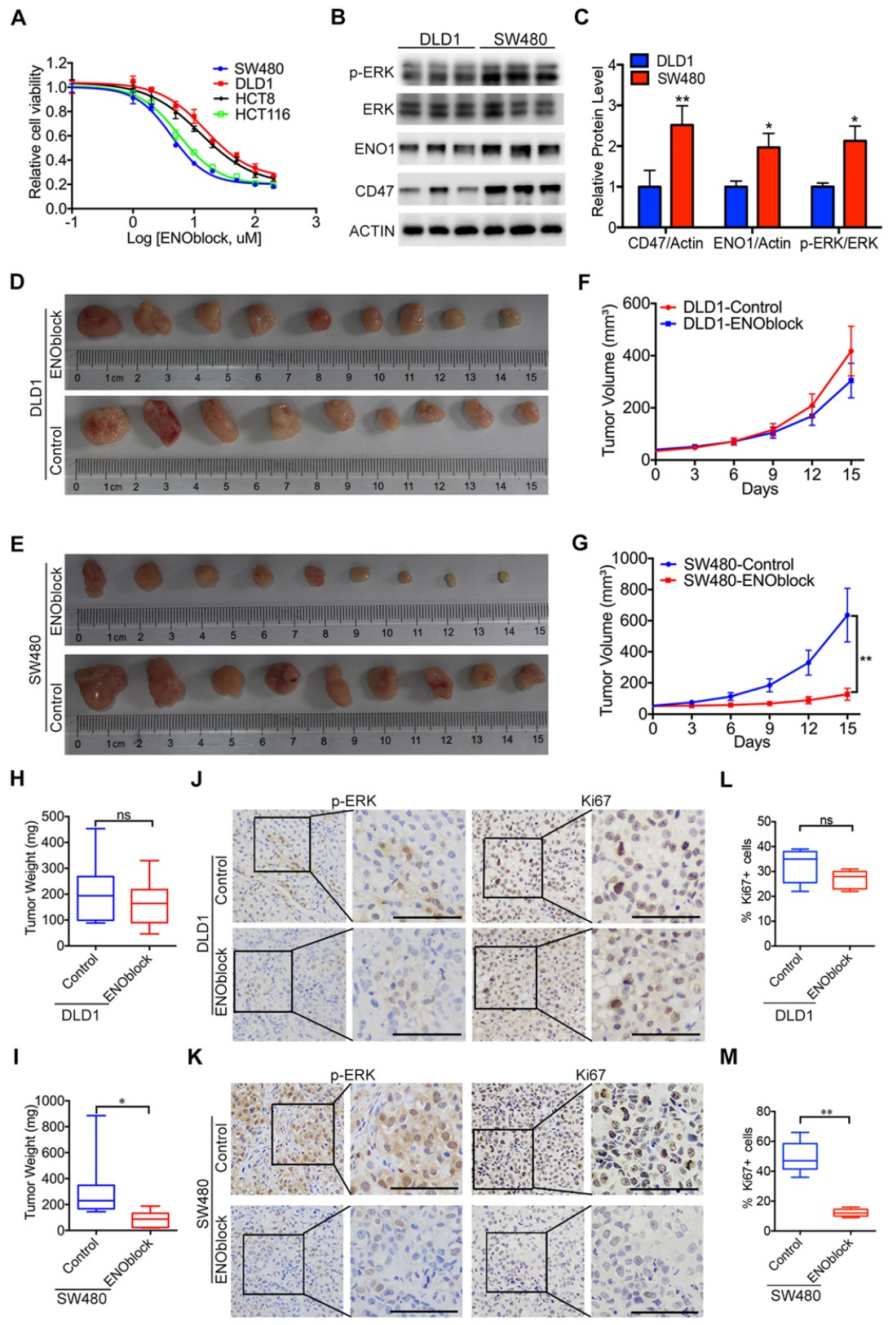
** Effectiveness of the ENO1 inhibitor in treating CD47-high tumors.** (A). The response of DLD1, HCT8, HCT116, and SW480 cells treated with different doses of ENOblock (100 nM-200 µM). (B-C). Immunoblotting and relative protein level of phosphorylated ERK (Thr202/Tyr204), ENO1 and CD47 in DLD1 and SW480 cells. (D-I). Representative images of tumors (D-E), statistics of tumor volume (F-G), and tumor weights (H-I) in nude mice bearing DLD1 and SW480 cells treated with DMSO or ENOblock (10 mg/kg/day, n=9 per group). Data are the means ± SD, *p<0.05, **p<0.01. (J-K). Representative IHC staining of p-ERK and Ki67 in nude mice bearing DLD1 or SW480 cells treated with DMSO or ENOblock. (L-M). Percentage of Ki67 positive cells in nude mice bearing DLD1 or SW480 cells treated with DMSO or ENOblock.

Since our results clearly showed that ENO1 is the functional mediator of CD47, blocking the CD47-ENO1 axis might be an effective treatment strategy for CD47-high tumors. CD47 antibody B6H12 did not show significant inhibitory effect on the cell proliferation of HCT116 and SW480 cells highly expressing CD47 (Figure S7A-B). We then sought to find a small molecule which can block the CD47-ENO1 axis to inhibit CRC cell proliferation. ENOblock is a newly identified inhibitor which can inhibit ENO activity in different cells and diseases models, including cancer 4, 31, 36. We treated CRC cell lines with varying doses of ENOblock (100nM-200μM) for 48h. CD47-high cells SW480 and HCT116 were more sensitive (IC50≈5μM) to ENOblock than CD47-low cells DLD1 and HCT8 (IC50≈40μM) (Figure 6A), suggesting that CRC cells with high expression of CD47 and ENO1 may be more sensitive to ENOblock. We found that low dose of ENOblock (5μM) treatment resulted in 50% of cell number decrease in SW480 (CD47 high) cells, but had no significant effect on DLD1 (CD47 low) cells (Figure 6B-C, Figure S7C-D). We further generated metabolite profiles using targeted metabolomics to determine the metabolic alterations in SW480 and DLD1 cells after ENO1 blockage. ENO block (5μM) induced a significant accumulation of 2-Phosphoglyceric acid (2PG, the substrate of ENO1), its upstream intermediates, and a reduction of PEP and other downstream metabolites in SW480 cells (Figure S7E). Nevertheless, 5μM ENOblock did not change the level of glycolytic metabolites in the DLD1 cells (Figure S7F). Most importantly, ENOblock treatment (10mg/kg) led to a remarkable decrease in tumor size and tumor weight in SW480 xenografts, while no significant effect was observed in DLD1 xenografts (Figure 6D-I). There were no substantial changes in body weight during ENOblock treatment (Figure S7G-H). IHC staining of tumor tissues showed that ENOblock treatment could significantly decrease the level of phosphorylated ERK and Ki67 in SW480 tumor tissues (Figure 6J, Figure 6L). However, these phenomena were not observed in DLD1 group (Figure 6K, Figure 6M). Together, our results suggest that targeting ENO1 might be an effective strategy for treating CD47-high tumors.

### Combination of CD47 and ENO1 provides a reliable prognostic maker for CRC

**Figure 7:**
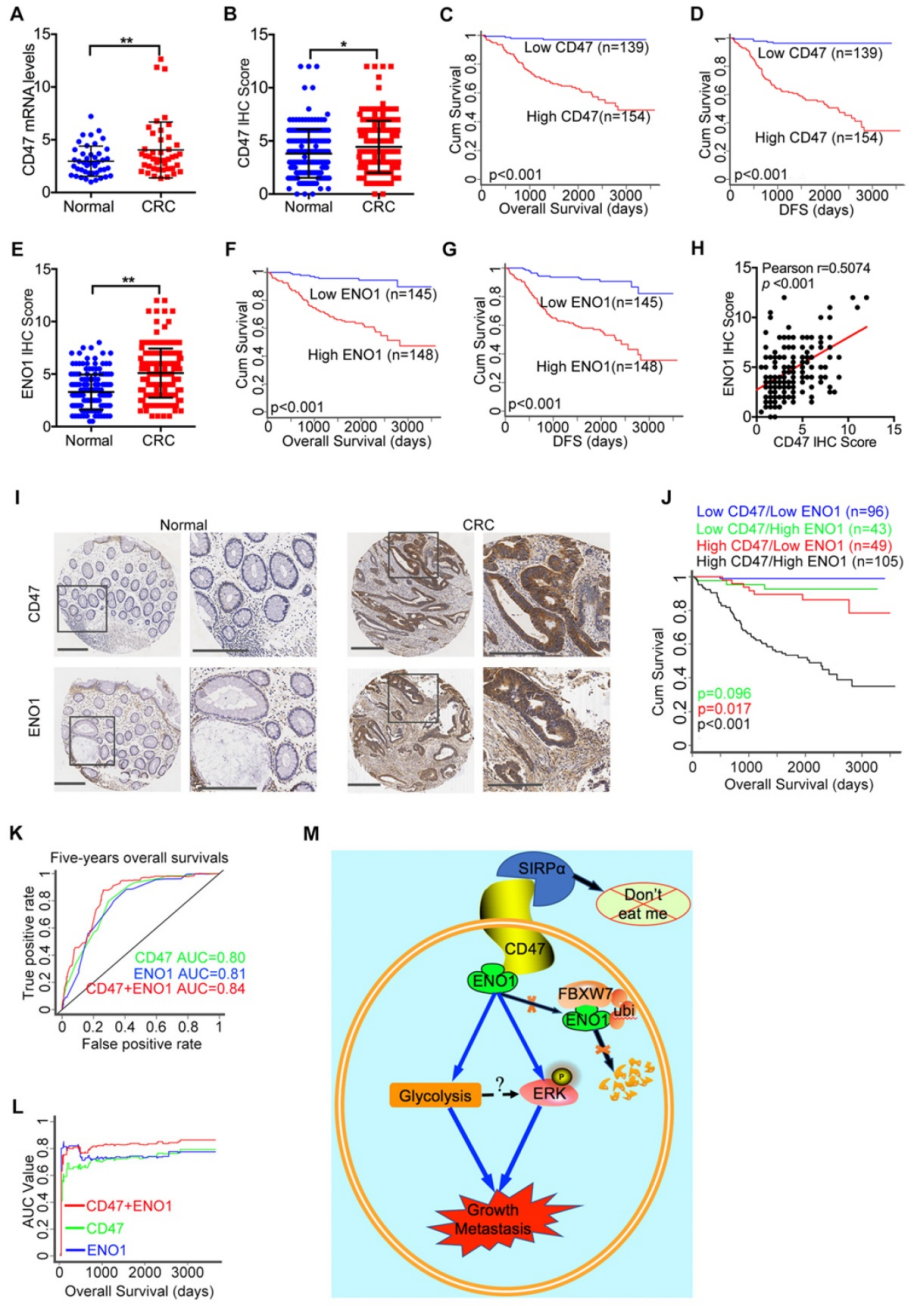
** High expression of CD47 and ENO1 predict poor survival outcomes of CRC patients.** (A). Statistical analysis of CD47 mRNA levels of 44 paired samples of CRC and adjacent normal tissues from the Sixth Affiliated Hospital (n = 44, non-parametric Wilcoxon matched-pairs signed rank test, *p*<0.01). (B). Scatter plot illustrating the protein level (IHC score) of CD47 in CRC and matched adjacent normal tissues. (n=179, *p*<0.05, Paired Student's t-test). (C-D). Survival analysis of CRC patients layered by the expression of CD47 in tissue microarrays from the Sixth Affiliated Hospital of Sun Yat-sen University (n = 293, Log-rank test, *p*<0.001, CD47-Low is defined as IHC score < 4 andCD47-High is defined as IHC score >= 4). (E). Scatter plot illustrating the protein level (IHC score) of ENO1 in CRC and matched adjacent normal tissues (n=179, *p*<0.01, Paired Student's t-test). (F-G). Survival analysis of CRC patients layered by the expression of ENO1 in tissue microarrays from the Sixth Affiliated Hospital of Sun Yat-sen University (n=293,* p*<0.001, ENO1-Low is defined as IHC score < 5 and ENO1-High is defined as IHC score >= 5). (H). Correlation analysis between CD47 and ENO1 protein levels (IHC score) in CRC samples from the Sixth Affiliated Hospital of Sun Yat-sen University. (n=293, Pearson's correlation, *p*<0.001). (I). Representative IHC staining of CD47 and ENO1 in human CRC tissues (right) and adjacent normal tissues (left). (J). Survival analysis of CRC patients layered by the expression of CD47 and ENO1 in tissue microarrays from the Sixth Affiliated Hospital of Sun Yat-sen University (n = 293, Log-rank test). (K-L). Receiver operating characteristic (ROC) curve and area under curve (AUC) values for the prognostic prediction model based on CD47 and ENO1 expression. (M). A schematic model of CD47 function in CRC. CD47 interacts with ENO1 and inhibits the FBXW7-mediated degradation of ENO1, thereby increasing ERK phosphorylation and glycolysis, ultimately promoting proliferation and metastasis of CRC.

Finally, we evaluated the mRNA and protein level of CD47 in CRC and matched adjacent normal tissue collected in our hospital (the Sixth Affiliated Hospital, Sun Yat-sen University). The mRNA levels of CD47 in CRC tissues were significantly higher (*p*<0.01) than the matched adjacent normal tissues (Figure 7A). The immunohistochemistry (IHC) staining of tissue microarrays (TMA) confirmed that CD47 protein levels were higher (*p*<0.05) in CRC tissues compared to the matched adjacent normal tissues (Figure 7B, Figure 7I). Moreover, Wilcoxon log-rank analysis indicated that high CD47 expression level was correlated to poor overall and disease-free survival in CRC patients (*p*<0.001, Figure 7C-D). Consistent with these, we found that the mRNA levels of CD47 were upregulated in CRC cohorts from both TCGA and Oncomine datasets (Figure S8A-B). Although the mRNA level of CD47 does not correlated with patient survival based on the data from TCGA database, high mRNA level of CD47 was associated with poor disease-free survival (*p*<0.01) in CRC patients from the GEO database (GSE14333, Figure S8C). Collectively, these data show that CD47 is overexpressed in CRC, and that a high level of CD47 is correlated with poor prognosis in patients.

To further elucidate the clinical significance and the interaction between CD47 and ENO1, we subjected the matched TMA for IHC staining of CD47 and ENO1. IHC scores indicated that CRC tissues exhibited significantly higher ENO1 protein levels (n=167, *p*<0.01) compared to the corresponding adjacent normal tissues (Figure 7E, Figure 7I). Wilcoxon log-rank analysis demonstrated that high expression level of ENO1 was associated with poor overall and disease-free survival (n=293,* p*<0.001) in CRC patients (Figure 7F-G). Most importantly, the IHC score of CD47 was significantly correlated with that of ENO1 (Figure 7H). In addition, Kaplan-Meier analysis showed that CD47^high^ENO1^high^ patients had the worst overall survival compared to CD47^high^ENO1^low^ and CD47^low^ENO1^high^ patients (Figure 7J). We also found that CD47 and ENO1 expression levels were positively correlated with metastasis, recurrence, and AJCC stage in CRC patients (Figure S8D-E and Table 1). Univariate and multivariate Cox regression analysis demonstrated that CD47 or ENO1 was an independent predictor for overall and disease-free survival of CRC patients (Figure S8F-G and Table 2-3), but receiver operating characteristic (ROC) curve and area under curve (AUC) values showed that the combination of CD47 and ENO1 have a better predictive performance than CD47 or ENO1 alone (Figure 7K-L). Together, these results verify that the CD47-ENO1 axis is upregulated in CRC, and the expression of CD47 and ENO1 can be used as predictive biomarkers for the prognosis of CRC patients.

## Discussion

CD47 is intensively studied as an immunosuppressive molecule that prevents macrophage-mediated phagocytosis and antigen presentation upon interaction with its receptor SIRPα on the macrophages. SIRPα is expressed on the macrophages and plays an inhibitory function when it binds to CD47. Upregulation of CD47 was observed in various cancer types and plays a vital role in escaping the immune surveillance of macrophages 9,19. Blocking the CD47-SIRPα interaction using anti-CD47 antibodies sheds a new light on the cancer immunotherapy 18,37,38. However, in the present study, we showed that CD47 can also regulate tumor progression in an immune-independent manner. Our results demonstrate that through increasing aerobic glycolysis and activating MAPK signaling, CD47 promotes the proliferation and metastasis of CRC cells both *in vitro* and *in vivo*.

Currently, numerous research studies are investigating the therapeutic effects of targeting CD47 by blocking the interaction between the extracellular domain of CD47 and SIRPα 15, 39. Phase I clinical trials have demonstrated that anti-CD47 therapy is effective and tolerable in certain solid human tumors and lymphomas 37, 38. Nevertheless, the clinical significance and functional role of CD47 is poorly defined in human CRC. Previous studies demonstrated that CD47 was associated with cancer stem cell markers and thus contributed to metastasis 23, 24. In line with these findings, our study identified CD47 as a prognostic-related marker that is upregulated in CRC. Patients with high expression of CD47 had a statistically significantly shorter overall and disease-free survival compared with patients with low CD47 expression. CD47 was also found to be positively correlated with metastasis, recurrence, and AJCC stage in CRC patients. Collectively, these data strongly indicate that the expression of CD47 could be a vital factor in regulating human CRC progression. Indeed, our results demonstrated that overexpression of CD47 promotes proliferation and metastasis in CRC cells.

Enhanced expression of CD47 was observed in various types of cancers, such as lung cancer, ovarian cancer, and leukemia 9-11. However, few studies focused on the tumor-intrinsic role of CD47 signals. Instead, most of the available articles were concentrated on the immune checkpoint function of the CD47-SIRPα axis. For the first time, our studies demonstrated that upregulation of CD47 can promote the proliferation and metastasis of CRC cells both *in vitro* and *in vivo*. More importantly, our co-IP and mass spectrometry data revealed that ENO1 was a downstream target of CD47 in the tumor cells. ENO1 is a key glycolytic enzyme that catalyzes the production of phosphoenolpyruvate (PEP) from 2-phosphoglycerate (2-PG), which plays a crucial role in aerobic glycolysis and facilitates the Warburg effect in cancer cells 40-42. Studies have shown that overexpression of ENO1 was correlated with poor prognosis in various types of cancer, including CRC 28, 32, 43-45. Except for regulating glycolysis, ENO1 was reported to enhance tumor growth and metastasis via activating AKT and ERK pathways in glioma 43. Interestingly, two recent studies reported that the increased glycolytic signal and lactic acid production could induce the activation of MAPK/ERK pathway in different disease settings 46,47, suggesting a potential interaction between ENO1 and MAPK signaling. Furthermore, ERK signaling and aerobic glycolysis was reported to promote CRC progression 48,49. Consistent with these findings, our results showed that CD47 enhanced glycolysis and ERK activation in an ENO1-dependent manner. We found that CD47 interacted with ENO1 and inhibit FBXW7-mediated ubiquitylation of ENO1. Knockdown of ENO1 blocked CD47-mediated cancer cell proliferation and metastasis. Notability, some recent work also suggested another well-known immune checkpoint molecule, PD-L1, have immune-independent and tumor-intrinsic functions. For instance, PD-L1 was shown to regulate proliferation and autophagy process in melanoma and ovarian cancer cells 50. High levels of PD-L1 inhibited cell apoptosis 51, increased chemo-resistance 52, and modulated glucose metabolism in sarcomas 53. These studies, including ours, extend our understanding of tumor cell immune checkpoint ligands, suggesting that more work is required to illustrate the non-immune effects of these molecules.

Aside from its biological significance, our data also showed that the status of CD47-ENO1 axis might be used for the treatment strategies determination and prognosis prediction in CRC patients. The survival analysis indicated that both CD47 and ENO1 could be independently used as a predictive biomarker of prognosis in CRC patients, but the combination of CD47 and ENO1 has a better predictive performance than CD47 or ENO1 alone in CRC patients. The CD47-ENO1 axis also presents as a promising therapeutic target in CRC patients. Specifically, we showed that pharmacological inhibition of ENO1 suppressed cell proliferation and tumor growth both *in vitro* and *in vivo*, but the ENOblock inhibitor was more effective in the CRC cells with higher level of CD47, indicating that CD47-high CRC patients might be more sensitive to ENO1 targeting therapy. Beside CRC, several studies illustrate that ENOblock could also effectively inhibit tumor metastasis in other cancer types 36,54.

In summary, our study illustrated that CD47 is overexpressed in human CRC tissues, and its upregulation is correlated with poor prognosis in CRC patients. Functionally, CD47 could promote cell proliferation and enhance the metastatic capability of CRC cells *in vitro* and *in vivo*. Mechanistically, CD47 could physically interact with ENO1 and prevent it from FBXW7-mediated ubiquitination, thereby increasing aerobic glycolysis and the activity of MAPK signaling. Given the clinical and biological significance of the CD47-ENO1 signaling axis, our data demonstrated that CD47 and ENO1 could be used as predictive biomarkers for prognosis and therapeutic targets in CRC. Furthermore, the varying expression levels of CD47 and ENO1 could be utilized to develop individual treatment plans in specific patients.

## Supplementary Material

Supplementary figures, tables, materials and methods.

## Figures and Tables

**Table 1:** Correlation between Expression of CD47, ENO1 and Clinicopathological Features in CRC Patients

Variables	Low CD47	High CD47	*p* value	Low ENO1	High ENO1	*p* value
Numbers	139	154		145	148	
**Gender**			0.229			0.887
Male	89 (64.0)	88 (57.1)		87 (60.0)	90 (60.8)	
Female	50 (36.0)	66 (42.9)		58 (40.0)	58 (39.2)	
**Median age**			0.070			0.070
<63 years	77 (55.4)	69 (44.8)		80 (55.2)	66 (44.6)	
≥63 years	62 (44.6)	85 (55.2)		65 (44.8)	82 (55.4)	
**BMI (kg/m^2^)**			0.330			0.403
<21.67	54 (46.2)	64 (52.5)		57 (46.7)	61 (52.1)	
≥21.67	63 (53.8)	58 (47.5)		65 (53.3)	56 (47.9)	
**pT stage**			0.330			0.186
T1	8 (5.8)	5 (3.3)		9 (6.2)	4 (2.7)	
T2	27 (19.4)	22 (14.3)		25 (17.2)	24 (16.2)	
T3	98 (70.5)	116 (75.3)		106 (73.1)	108 (73.0)	
T4	6 (4.3)	11 (7.1)		5 (3.5)	12 (8.1)	
**pN stage**			0.464			0.774
N0	86 (61.9)	87 (56.5)		86 (59.3)	87 (58.8)	
N1	37 (26.6)	42 (27.3)		37 (25.5)	42 (28.4)	
N2	16 (11.5)	25 (16.2)		22 (15.2)	19 (12.8)	
**pM stage^a^**			0.002			0.001
M0	135 (97.1)	133 (86.4)		141 (97.2)	127 (85.8)	
M1	4 (2.9)	21 (13.6)		4 (2.8)	21 (14.2)	
**TNM stage**			0.004			0.004
I	32 (23.0)	21 (13.6)		30 (20.7)	23 (15.5)	
II	53 (38.1)	58 (37.7)		55 (37.9)	56 (37.8)	
III	50 (36.0)	54 (35.1)		56 (38.6)	48 (32.4)	
IV	4 (2.9)	21 (13.6)		4 (2.8)	21 (14.2)	
**Histological grade**			0.801			0.439
G1	13 (9.4)	17 (11.0)		18 (12.4)	12 (8.1)	
G2	104 (74.8)	116 (75.3)		105 (72.4)	115 (77.7)	
G3	22 (15.8)	21 (13.6)		22 (15.2)	21 (14.2)	
**Tumor size (cm)**			0.922			0.867
<5	66 (47.5)	74 (48.1)		70 (48.3)	70 (47.3)	
≥5	73 (52.5)	80 (51.9)		75 (51.7)	78 (52.7)	
**Recurrence**			<0.001			0.010
YES	4 (4.1)	42 (37.8)		12 (11.9)	34 (31.5)	
NO	94 (95.9)	69 (62.2)		89 (88.1)	74 (68.5)	
**CEA (ng/ml)**			0.638			0.249
<5	96 (73.3)	99 (70.7)		105 (75.0)	90 (68.7)	
≥5	35 (26.7)	41 (29.3)		35 (25.0)	41 (31.3)	
**CA199 (U/ml)**			0.538			0.074
<37	101 (78.3)	109 (81.3)		116 (84.1)	94 (75.2)	
≥37	28 (21.7)	25 (18.7)		22 (15.9)	31 (24.8)	
**Location**			0.797			0.258
colon	62 (44.6)	71 (46.1)		61 (42.1)	72 (48.6)	
rectum	77 (55.4)	83 (53.9)		84 (57.9)	76 (51.4)	

Note: All data are shown as numbers and percentages (%). CD47-Low is defined as IHC score < 4 and CD47-High is defined as IHC score ≥ 4. ENO1-Low is defined as IHC score < 5 and ENO1-High is defined as IHC score >= 5. a: The Yate's correction for continuity was used in data of this group.

**Table 2:** Univariate and Multivariate Analysis for the Prognostic Factors of Overall Survival in CRC Patients

Variables	Univariate Analysis HR (95% CI)	*p* value	Multivariate Analysis HR (95% CI)	*p* value
Gender (male versus female)	0.843 (0.520-1.367)	0.489	-	-
Age (≥63 years vs <63 years)	2.153 (1.292-3.587)	0.003	1.057 (0.537-2.081)	0.873
BMI (≥21.67 kg/m^2^ vs <21.67kg/m^2^)	1.020 (0.582-1.787)	0.944	-	-
pT status (T3-T4 vs T1-T2)	1.637 (0.836-3.207)	0.151	-	-
pN status (N1 vs N0)	1.765 (1.092-2.853)	0.020	4.985 (1.050-23.677)	0.043
pM status (M1 vs M0)	6.600 (3.821-11.400)	<0.001	8.142 (3.007-22.047)	<0.001
AJCC stage (III- IV vs I- II)	2.128 (1.306-3.469)	0.002	1.881 (1.343-6.383)	0.013
Histological grade (G3 vs G1-G2)	2.157 (1.226-3.794)	0.008	3.916 (1.762-8.703)	0.001
Tumor size (≥5 cm vs <5cm)	1.145 (0.705 -1.860)	0.584	-	-
Recurrence (Yes vs No)	5.078 (2.908-8.867)	<0.001	1.814 (0.882-3.732)	0.106
CD47 expression (High vs Low)	15.865 (5.771-43.611)	<0.001	35.854 (4.563-281.726)	0.001
ENO1 expression (High vs Low)	8.585 (4.101-17.972)	<0.001	3.770 (1.536-9.252)	0.004
CEA (≥5 ng/ml vs <5ng/ml)	2.057 (1.224-3.455)	0.006	3.109 (1.509-6.406)	0.002
CA199 (≥37 U/ml vs <37U/ml)	1.333 (0.716-2.482)	0.365	-	-
Location (Rectum vs Colon )	0.808 (0.509-1.282)	0.365	-	-

Univariate and multivariate Cox proportional hazards regression were used to calculate Hazard ratio (HR), 95% confidence intervals (95% CI) and p values in SPSS 22.0. CD47-Low is defined as IHC score < 4 andCD47-High is defined as IHC score >= 4. ENO1-Low is defined as IHC score < 5 and ENO1-High is defined as IHC score ≥ 5.

**Table 3:** Univariate and Multivariate Analysis for the Prognostic Factors of Disease-Free Survival in CRC Patients

Variables	Univariate Analysis HR (95% CI)	*p* value	Multivariate Analysis HR (95% CI)	*p* value
Gender (male versus female)	0.843 (0.549-1.295)	0.436	-	-
Age (≥63 years vs <63 years)	1.657 (1.071-2.565)	0.023	1.310 (0.782-2.197)	0.305
BMI (≥21.67 kg/m^2^ vs <21.67kg/m^2^)	1.024 (0.622-1.686)	0.926	-	-
pT status (T3 or T4 vs T1 or T2)	1.851 (1.004-3.411)	0.048	0.959 (0.422-2.178)	0.920
pN status (N1 vs N0)	1.783 (1.164-2.730)	0.008	4.604 (1.680-12.616)	0.003
pM status (M1 vs M0)	6.123 (3.719-10.081)	<0.001	3.161 (1.376-7.261)	0.007
AJCC stage (III or IV vs I or II)	2.204 (1.427-3.403)	<0.001	3.928 (1.497-10.039)	0.005
Histological grade (G3 vs G1 or G2)	1.763 (1.045-2.972)	0.033	2.262 (1.192-4.291)	0.012
Tumor size (≥5 cm vs <5cm)	1.314 (0.852-2.027)	0.216	-	-
Recurrence (Yes vs No)	15.547 (9.115-26.157)	<0.001	7.922 (4.225-14.854)	<0.001
CD47 expression (High vs Low)	17.264 (6.990-42.639)	<0.001	6.848 (2.365-19.829)	<0.001
ENO1 expression (High vs Low)	6.179 (3.480-10.969)	<0.001	3.104 (1.602-6.017)	0.001
CEA (≥5 ng/ml vs <5ng/ml)	1.445 (0.893-2.339)	0.134		
CA199 (≥37 U/ml vs <37U/ml)	1.152 (0.651-2.039)	0.627	-	-
Location (Rectum vs Colon )	0.829 (0.550-1.248)	0.369	-	-

Univariate and multivariate Cox proportional hazards regression were used to calculate Hazard ratio (HR), 95% confidence intervals (95% CI) and p values in SPSS 22.0. CD47-Low is defined as IHC score < 4 andCD47-High is defined as IHC score >= 4. ENO1-Low is defined as IHC score < 5 and ENO1-High is defined as IHC score ≥ 5.

## Abbreviations

FC): Immunohistochemistry (IHC
LC-MS/MS): Phosphoenol pyruvate (PEP
