# Supplementary material for: Tumor-intrinsic CD47 signal regulates glycolysis and promotes colorectal cancer cell growth and metastasis
Source: Theranostics. 2020 Mar 4;10(9):4056–72. doi: 10.7150/thno.40860 (PMC7086360; doi:10.7150/thno.40860)
Supplement: Supplementary file 1 — Supplementary figures, tables, materials and methods. [file thnov10p4056s1.pdf]

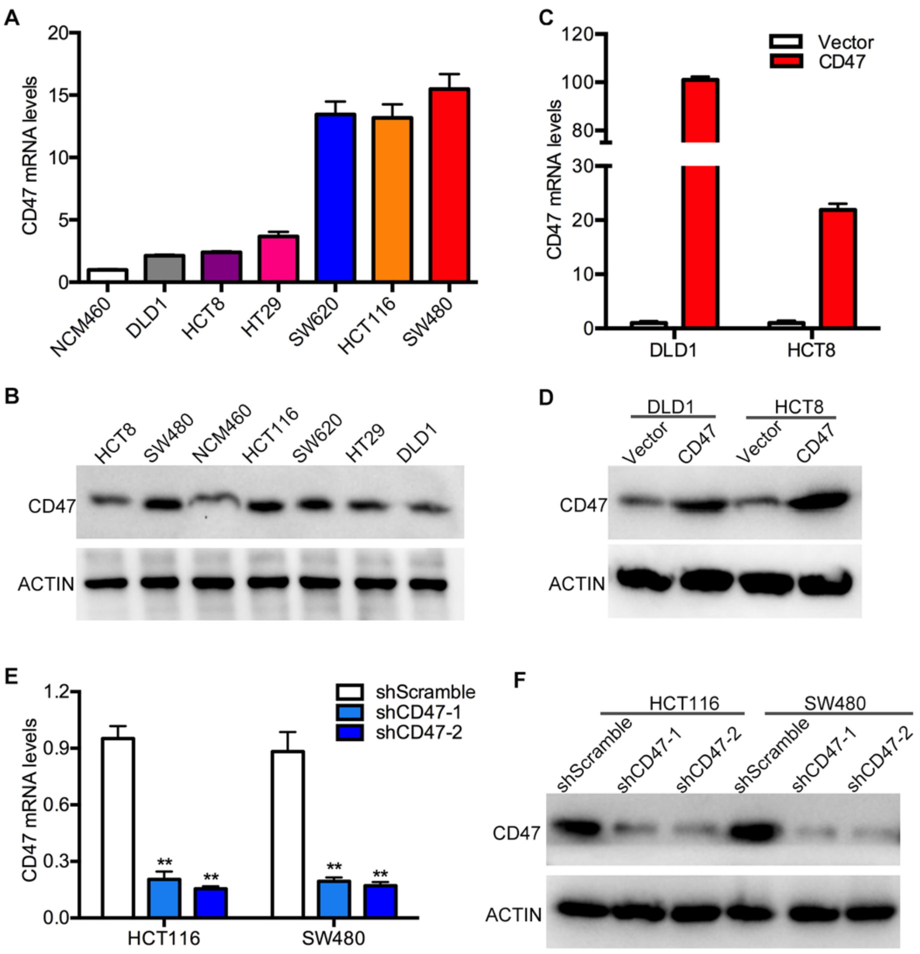

Figure S1. Validation of CD47 over-expression and knockdown efficiency in CRC cell lines. (A). CD47 mRNA levels in CRC cell lines measured by qRT-PCR. Data are the means ± SD. (B). Immunoblotting of CD47 expression levels in CRC cell lines. (C-D). qRT-PCR and western blot assays used for the detection of CD47 expression in HCT8 and DLD1 cells stably overexpressing CD47. (E-F). qRT-PCR and western blot assays used for the detection of CD47 expression in stable CD47 knockdown HCT116 and SW480 cells. Data are the means ± SD, **p*<0.05, ***p*<0.01 and ****p*<0.001.

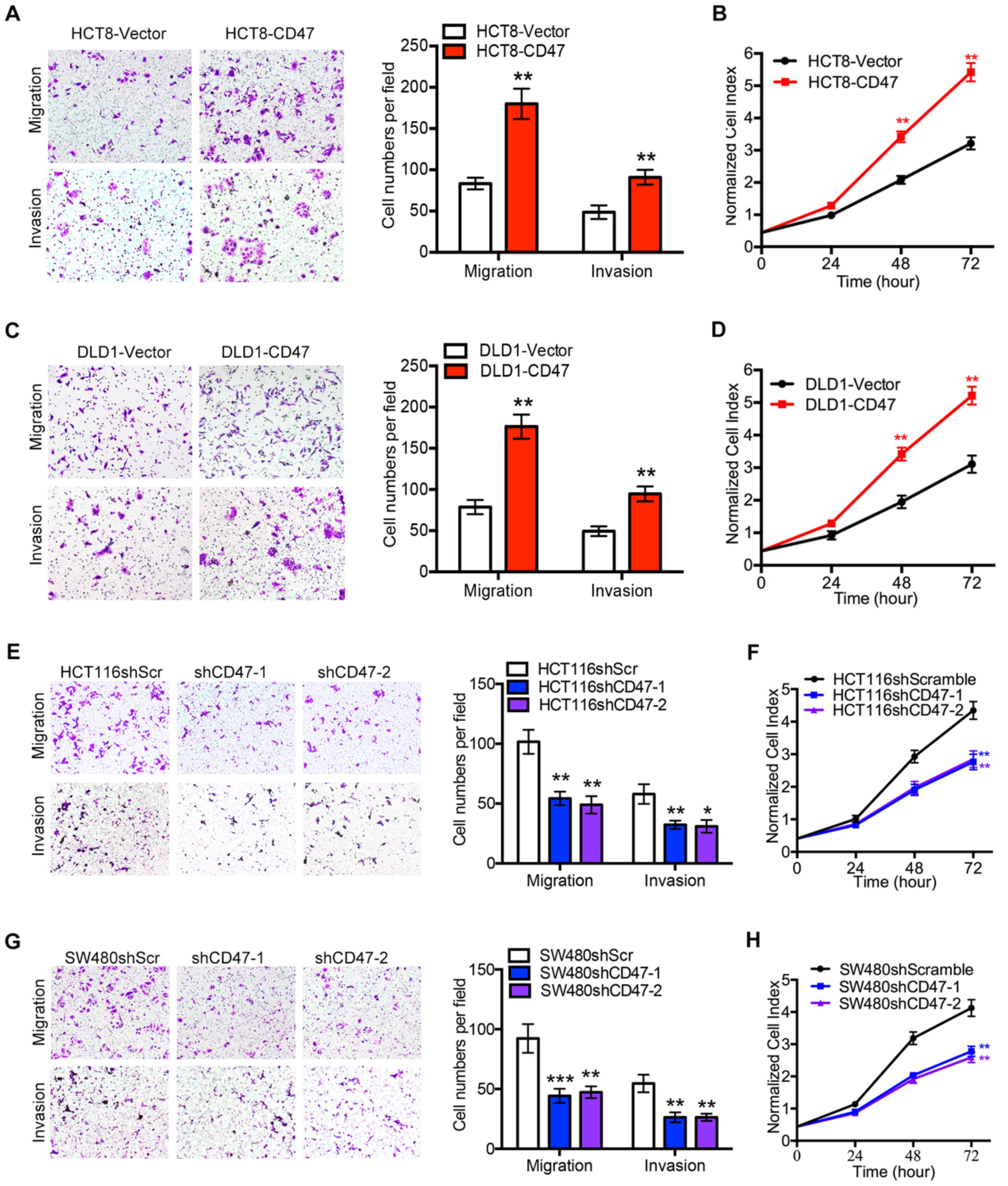

Figure S2. CD47 enhances CRC cell proliferation, migration, and invasion *in vitro*. (A-D). Transwell migration /invasion assays and cell proliferation assays performed in HCT8 and DLD1 cells stably overexpressing CD47. (E-H). Transwell migration/invasion assays and cell proliferation assays conducted in stable CD47 knockdown HCT116 and SW480 cells. Data are the means ± SD, *p<0.05, **p<0.01, ***p<0.001.

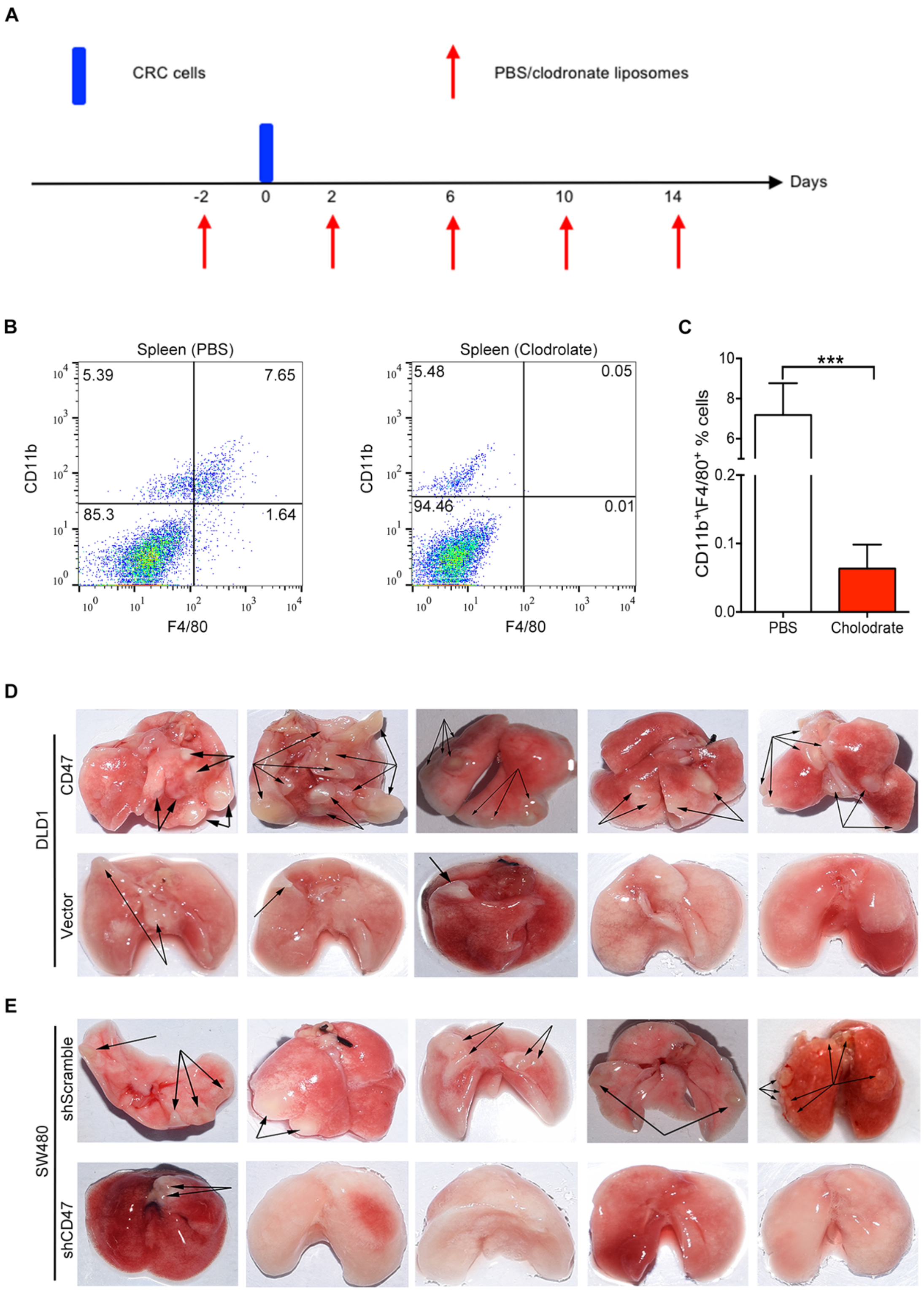

Figure S3. Clodronate liposomes-mediated macrophage depletion in vivo. (A). Schematic outline of the macrophage depletion induced by clodronate liposomes. Nude mice were administered either PBS liposomes or clodronate liposomes (0.25 mL/mouse) by intraperitoneal (i.p.) injection at day -1, 4, 8,12 and 16.
(B-C). Splenocytes isolated from the nude mice in each group were stained with CD11b and F4/80 antibodies. The macrophage depletion efficiency was determined by the relative percentage of F4/80+/CD11b+ cells.
(D-E). DLD1-Vector, DLD1-CD47, SW480-shScramble, and SW480-shCD47 cells were injected into tail veins of nude mice (n = 5). Representative images of lung metastatic tumors are shown. Data are the means ± SD. **p*<0.05, ***p*<0.01, ****p*<0.001.

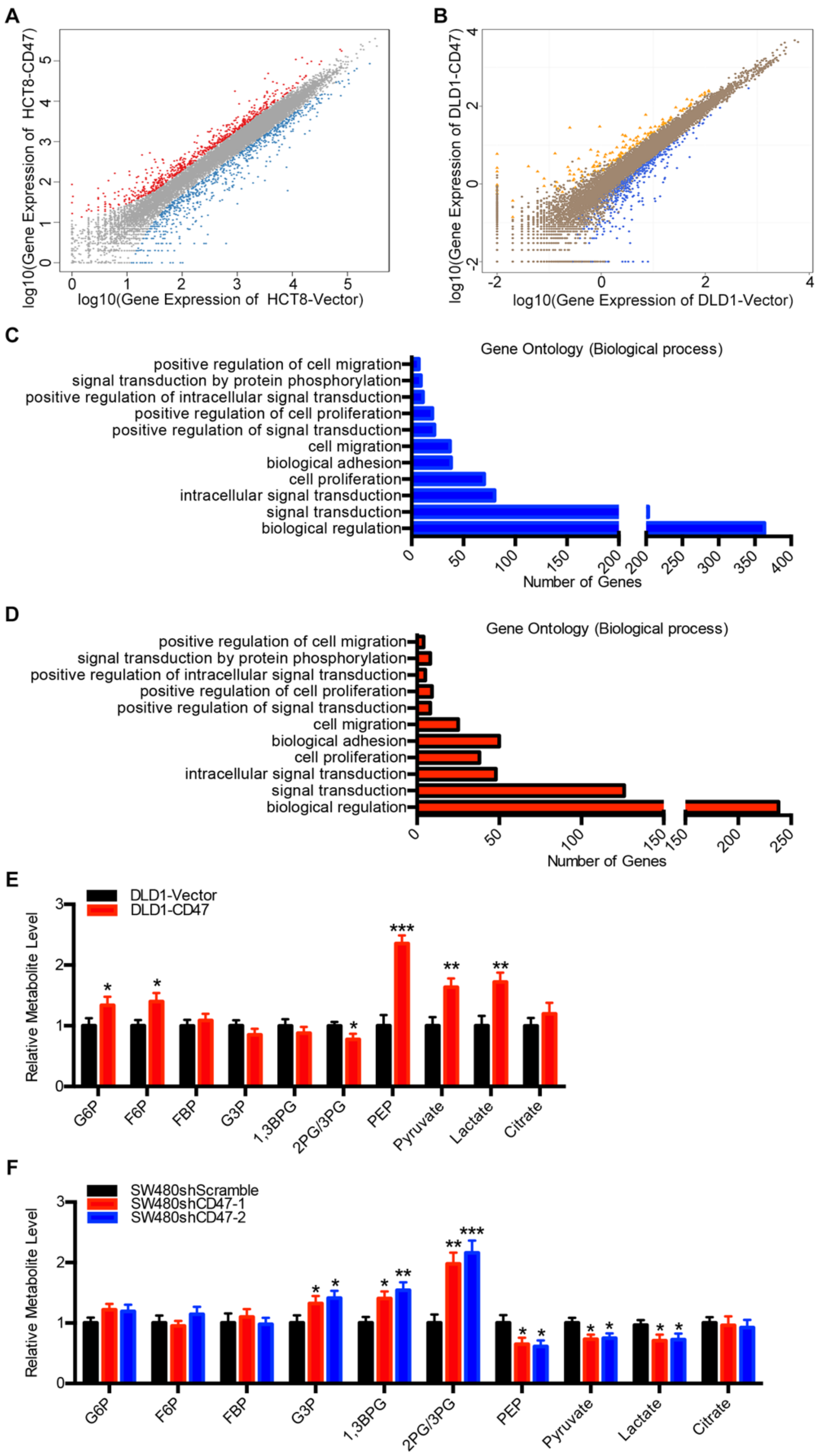

Figure S4. RNA-seq analysis in CD47-overexpressed CRC cells. (A-B). Scatter-plot of RNA sequencing data showing differentially expressed genes (fold change>2 and FDR<0.001) regulated by CD47 over-expression in HCT8 and DLD1 cells. (C-D). Functional annotation of genes regulated by CD47 over-expression in HCT8 and DLD1 cells. Enriched gene groups were listed by their gene ontology term. (E). Relative metabolite levels in glycolysis and TCA cycle from DLD1-Vector and DLD1-CD47 cells. (F). Relative metabolite levels in glycolysis and TCA cycle from SW480shScramble and SW480shCD47 cells.

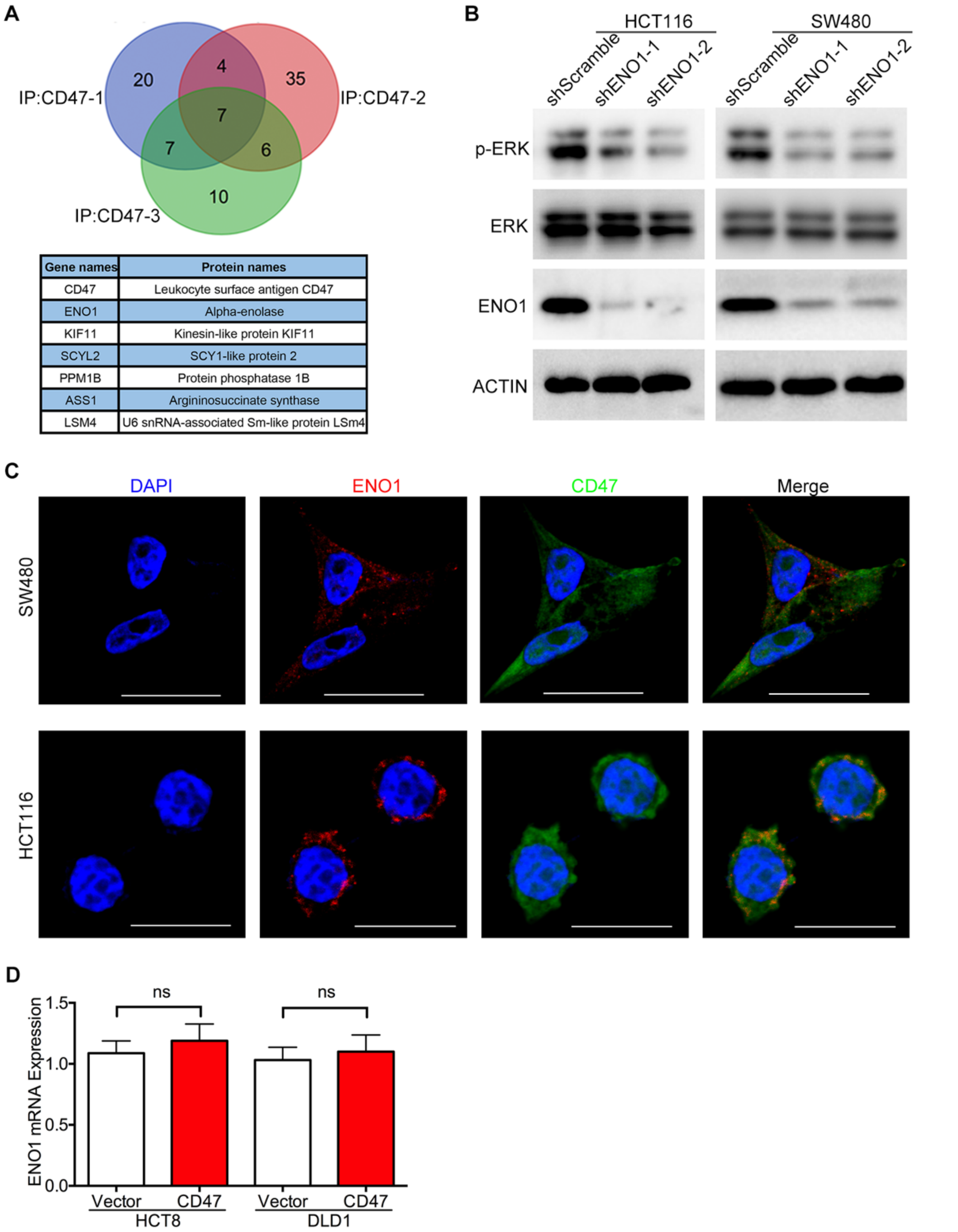

Gene names	Protein names
CD47	Leukocyte surface antigen CD47
ENO1	Alpha-enolase
KIF11	Kinesin-like protein KIF11
SCYL2	SCY1-like protein 2
PPM1B	Protein phosphatase 1B
ASS1	Argininosuccinate synthase
LSM4	U6 snRNA-associated Sm-like protein LSm4

Figure S5. CD47 enhances aerobic glycolysis and activates ERK pathway via ENO1 upregulation. (A). Venn diagram depicting CD47-associated proteins from three independent immunoprecipitation and mass spectrometry experiments. (B). Immunoblotting of ENO1 and ERK (Thr202/Tyr204) phosphorylation in HCT116 and SW480 cells transfected with ENO1-targeting shRNA. (C). Immunofluorescence illustrating CD47 co-localization with ENO1 in HCT8 and DLD1 cells, Scale bars represent 20 μm. (D). ENO1 mRNA levels in CD47-overexpressed HCT8 and DLD1 cells.

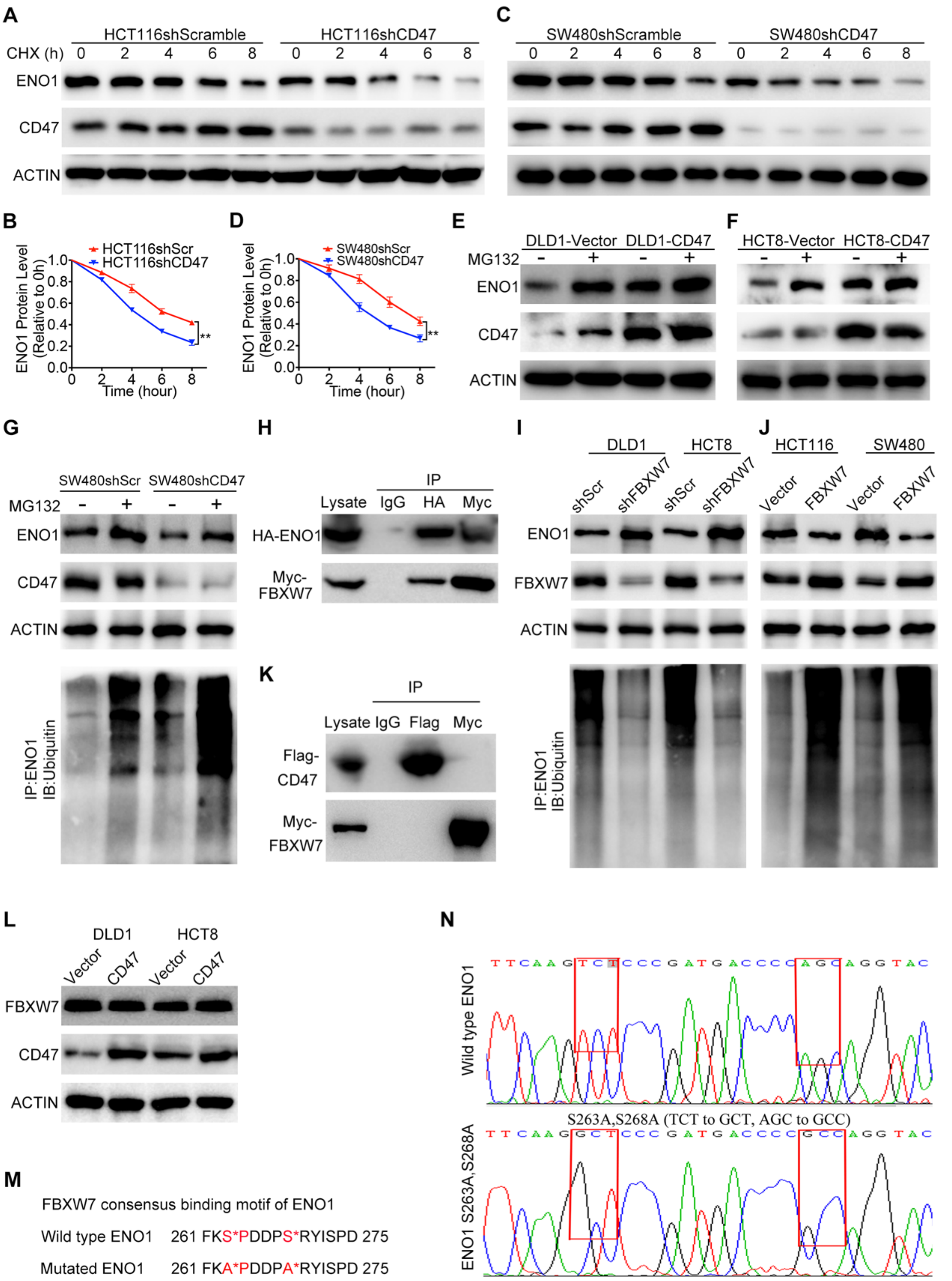

Figure S6. CD47 inhibits FBXW7-mediated ubiquitylation and degradation of ENO1. (A-B). Immunoblotting and quantitative analysis for ENO1 protein levels in CD47 knockdown HCT116 cells treated with CHX (50 μg/mL). (C-D). Immunoblotting and quantitative analysis for ENO1 protein levels in CD47 knockdown SW480 cells treated with CHX (50 μg/mL). Data are the means ± SD. (E-F). Immunoblotting for ENO1 in CD47-overexpressed DLD1 and HCT8 cells treated with MG132 (25μM) for 12 h. (G). ENO1 protein level and endogenous ENO1 ubiquitination level in control and CD47 knockdown SW480 cells treated with MG132 (25μM) for 12 h. (H). HEK293T cells were transfected with Myc-FBXW7 and HA-ENO1 plasmids and MG132 (25μM) was added 6h before harvest. Cell lysates were immunoprecipitated with anti-Myc/HA magnetic beads and then immunoblotted with the indicated antibodies. (I-J). Endogenous ENO1 ubiquitylation level in FBXW7-knockdown DLD1/HCT8 cells and FBXW7-overexpressed HCT116/SW480 cells. (L). Immunoblotting for FBXW7 in CD47-overexpressed HCT8 and DLD1 cells. (K). Cell lysates of HEK293T cells transfected with Flag-CD47 and Myc-FBXW7 plasmids were immunoprecipitated with anti-Flag/Myc magnetic beads and then immunoblotted with the indicated antibodies. (M). FBXW7 consensus binding motif of ENO1. (N). Sequencing results of wild type ENO1 and mutant ENO1 (S263A/S268A) plasmids.

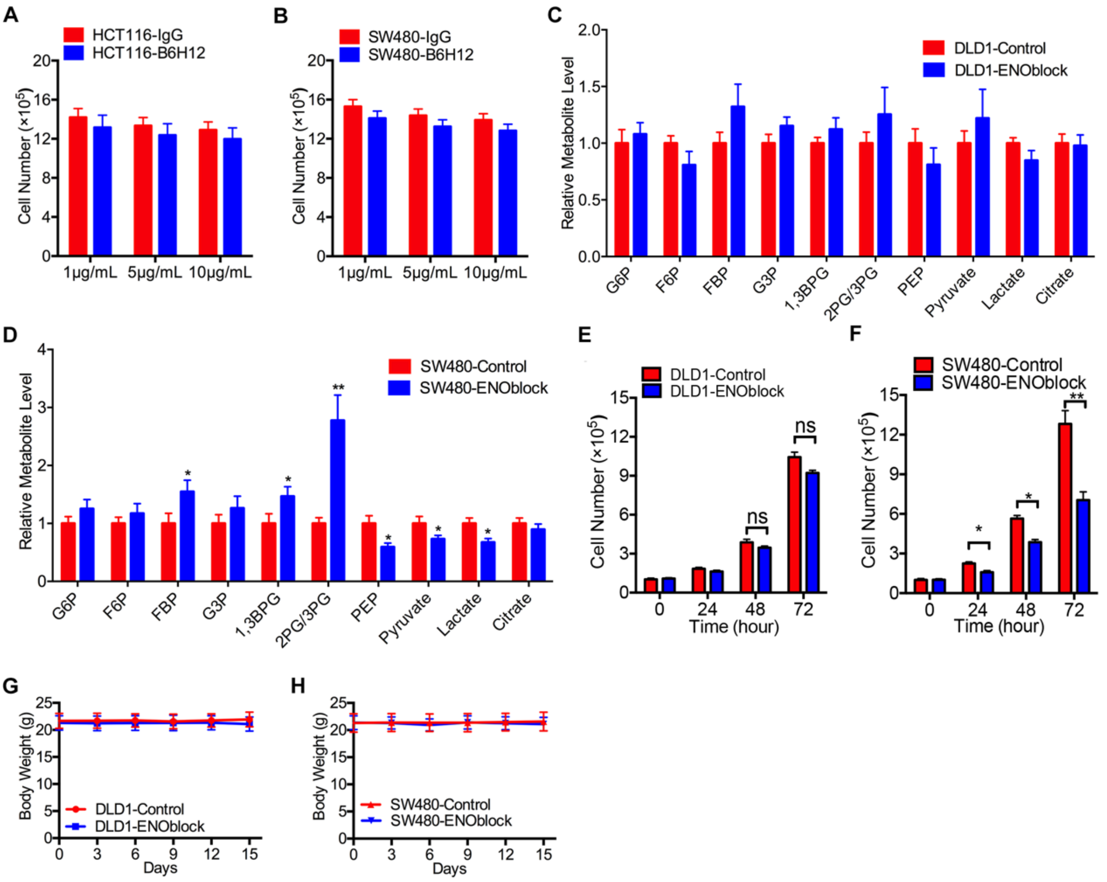

Figure S7. Effectiveness of ENO1 inhibitor in treating CD47-high tumors. (A-B). Cell counting assays performed in SW480 and HCT116 cells treated with CD47 antibody B6H12 (1, 5, 10 μg/mL, BioXcell, West Lebanon, USA) for 48 h. (C-D). Cell counting assays performed in DLD1 and SW480 cells treated with 5Mm ENOblock (n=3, non-parametric Mann-Whitney test, **p*<0.05, ***p*<0.01). (E-F). Relative levels of glycolytic and TCA cycle metabolites in DLD1 and SW480 cells treated with DMSO or ENOblock (5μM). (G-H). Body weight of nude mice inoculated with DLD1 and SW480 cells treated with DMSO or ENOblock (n=9 per group).

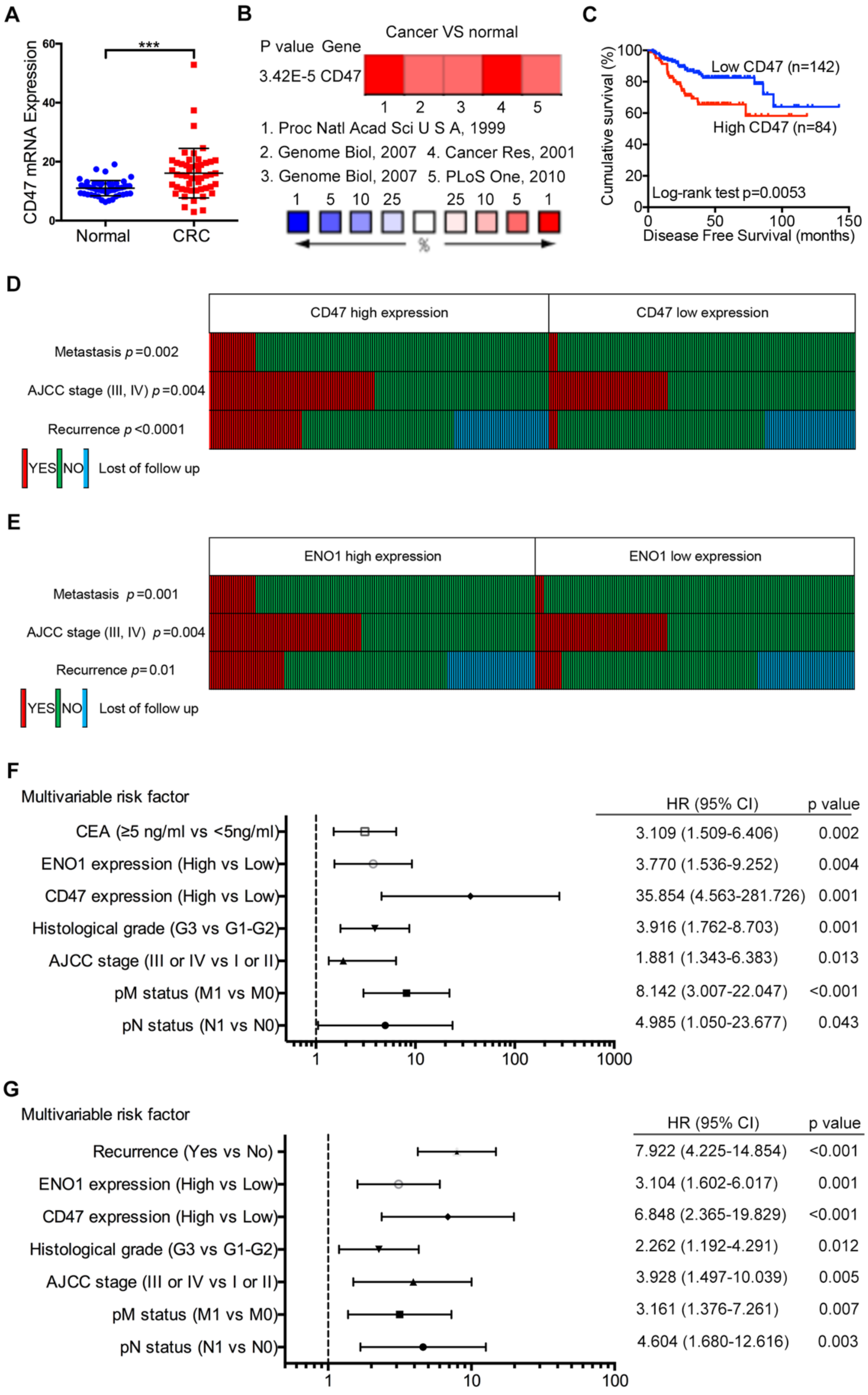

Figure S8. The clinical relevance of CD47 and ENO1 in CRC. (A). CD47 mRNA levels in CRC and the paired adjacent normal tissues from TCGA database (n = 50, non-parametric Wilcoxon matched-pairs signed rank test, p < 0.001). (B). CD47 is overexpressed in CRC tissues among various cohorts from the Oncomine database. (C). High expression of CD47 mRNA is associated with poor disease-free survival in GEO cohort (GSE14333, n=226, Log-rank test, p = 0.0053, CD47 expression level less than 7.90 is defined as low. CD47 expression level more than 7.90 is defined as high.). (D-E). A heat map demonstrating the relationship between clinicopathological features and CD47 and ENO1 expression (n = 293, Chi-square test). (F). Multivariate regression analysis of overall survival time (OS) in CRC patients (Bars indicate 95% confidence intervals). (G). Multivariate regression analysis of disease-free survival time (DFS) in CRC patients (Bars indicate 95% confidence intervals).

Supplementary Tables

Table S1. Antibodies and beads used in this study.

Name	Company	Catalog Number	Assay
ENO1	Abcam	# ab227978	IP,WB,IHC
CD47	Abcam	# ab175388	WB ,IHC,IF
CD47 (B6H12)	eBioscienceTM	#14-0479-82	IP
CD47 (B6H12)	BioXcell	#BE0019-1	Cell assay
FBXW7	Abcam	#ab109617	WB
p38	Cell Signaling Technology	#9212	WB
P-p38 (Thr180/Tyr182)	Cell Signaling Technology	#9211	WB
SAPK/JNK	Cell Signaling Technology	#9252	WB
P-SAPK/JNK (Thr183/Tyr185)	Cell Signaling Technology	#9251	WB
ERK	Cell Signaling Technology	#4695	WB
P-ERK(thr202/tyr204)	Cell Signaling Technology	#4370	WB,IHC
β-actin	Sigma-aldrich	#A3854	WB
Ubiquitin	Cell Signaling Technology	#3933	WB
Anti-HA antibody	Sigma-aldrich	#H9658	WB
Anti-FLAG® M2 antibody	Sigma-aldrich	#F3165	WB
Anti-Myc-Tag antibody	Bimake	#A5968	WB
Anti-Flag magnetic beads	Bimake	#B26101	IP
Anti-HA magnetic beads	Bimake	#B26201	IP
Anti-Myc magnetic beads	Bimake	#B26301	IP
Rabbit mAb IgG XP® Isotype Control	CST	#8726	IP

Table S2. The enriched signaling pathways and biological processes for CD47 over-expression.

KEGG (Pathway Enrichment, HCT8-CD47 VS HCT8-Vector)

Pathway Name	Pathway ID	Pvalue	Qvalue
TGF-beta signaling pathway	ko04350	5.69E-05	0.00612802
Glycine, serine and threonine metabolism	ko00260	2.50E-04	0.016397633
Biosynthesis of amino acids	ko01230	2.74E-04	0.016397633
IL-17 signaling pathway	ko04657	0.001622381	0.052402906
MAPK signaling pathway	ko04010	0.002101148	0.054572217
TNF signaling pathway	ko04668	0.00792529	0.140771016
Metabolic pathways	ko01100	0.008280648	0.140771016
Glycolysis / Gluconeogenesis	ko00010	0.03221753	2.70E-01

KEGG (Pathway Enrichment, DLD1-CD47 VS DLD1-Vector)

Pathway Name	Pathway ID	Pvalue	Qvalue
Serotonergic synapse	ko04726	0.000355148	0.09198336
MAPK signaling pathway	ko04010	0.003697463	0.31829607
Inflammatory mediator regulation of TRP channels	ko04750	0.004165514	0.31829607
ECM-receptor interaction	ko04512	0.006726048	0.31829607
Ovarian steroidogenesis	ko04913	0.009487112	0.31829607
Cell adhesion molecules (CAMs)	ko04514	0.01102188	0.31829607
alpha-Linolenic acid metabolism	ko00592	0.01578699	0.31829607
Glycolysis / Gluconeogenesis	ko00010	0.03708984	0.40026119

Gene Ontology (Biological Process, HCT8-CD47 VS HCT8-Vector)

Gene Ontology term	GO_ID	Cluster frequency
biological regulation	GO:0065007	363 of 699 in the list
biological adhesion	GO:0022610	38 of 699 in the list
cell migration	GO:0016477	37 of 699 in the list
cell proliferation	GO:0008283	70 of 699 in the list
positive regulation of cell migration	GO:0030335	7 of 699 in the list
positive regulation of cell proliferation	GO:0008284	20 of 699 in the list
positive regulation of signal transduction	GO:0009967	22 of 699 in the list
signal transduction	GO:0007165	203 of 699 in the list
intracellular signal transduction	GO:0035556	80 of 699 in the list
positive regulation of intracellular signal transduction	GO:1902533	11 of 699 in the list
signal transduction by protein phosphorylation	GO:0023014	9 of 699 in the list

Gene Ontology (Biological Process, DLD1-CD47 VS DLD1-Vector)

Gene Ontology term	GO_ID	Cluster frequency
biological regulation	GO:0065007	238 of 445 in the list
biological adhesion	GO:0022610	50 of 445 in the list
cell migration	GO:0016477	25 of 445 in the list
cell proliferation	GO:0008283	38 of 445 in the list
positive regulation of cell migration	GO:0030335	4 of 445 in the list
positive regulation of cell proliferation	GO:0008284	9 of 445 in the list
signal transduction	GO:0007165	126 of 445 in the list
intracellular signal transduction	GO:0035556	48 of 445 in the list
positive regulation of intracellular signal transduction	GO:1902533	5 of 445 in the list
positive regulation of signal transduction	GO:0009967	8 of 445 in the list
signal transduction by protein phosphorylation	GO:0023014	8 of 445 in the list

Table S3. Primers and siRNA sequence used in this study.

**Primers used for quantitative qRT-PCR**		
**Name**	**Forward-primer**	**Reverse-primer**
CD47	AGTGATGGACTCCGATTTGG	GGGTCTCATAGGTGACAACCA
ENO1	TGGTGTCTATCGAAGATCCCTT	CCTTGGCGATCCTCTTTGG
β-actin	GTCATTCCAAATATGAGATGCGT	GCATTACATAATTTACACGAAAGCA
**Primers used for plasmid construction**		
**Name**	**Forward-primer**	**Reverse-primer**
CD47-CDS	CCCGGACGAATTC TTCGAA ACCATG ATG TGG CCC CTG GTA GCG	TGCGGATCACTAGT GCTAGC TTATTCATCATTCATCATTCC
Flag-CD47	CCCGGACGAATTC TTCGAA ACCATG ATG GAC TAC AAA GAC GAT GAC GAT AAA ATG TGG CCC CTG GTA GCG	TGCGGATCACTAGT GCTAGC TTATTCATCATTCATCATTCC
ENO1-CDS	CCCGGACGAATTC TTCGAA ATG TCT ATT CTC AAG ATC CAT GCC	TGCGGATCACTAGTGCTAGCTTACTTGG CCAAGGGGTTTCT GAAG
HA-ENO1	CCCGGACGAATTC TTCGAA ATG TAC CCA TAC GAT GTT CCA GAT TAC GCT ATG TCT ATT CTC AAG ATC CAT GCC	TGCGGATCACTAGT GCTAGC TTA CTT GGC CAA GGG GTT TCT GAA G
Myc-FBXW7	CCCGGACGAATTC TTCGAA ATG GAG CAG AAA CTC ATC TCT GAA GAG GAT CTG ATG AAT CAG GAA CTG CTC TCT GTG	TGCGGATCACTAGT GCTAGC TCA CTT CAT GTC CAC ATC AAA GTC
**Primers used for shRNA construction**		
**Name**	**Forward-primer**	**Reverse-primer**
shCD47-1	CCGG— AAACGATCATCGAGCTAAA — CTCGAG— TTTAGCTCGATGATCGTTT —TTTTTG	AATTCAAAAA— AAACGATCATCGAGCTAAA — CTCGAG—TTTAGCTCGATGATCGTTT
shCD47-2	CCGG— GGCGTGTATACCAATGCAT— CTCGAG—ATGCATTGGTATACACGCC —TTTTTG	AATTCAAAAA— GGCGTGTATACCAATGCAT—CTCGAG— ATGCATTGGTATACACGCC
**Primers used for mutation plasmid construction**		
**Name**	**Forward-primer**	**Reverse-primer**
mutant HA-ENO1 (S263)	GCT CCC GAT GAC CCC AGC AGG TAC ATC	CTT GAA GTC CAG GTC ATA CTT CCC AG
mutant HA-ENO1 (S263A/S268A)	GCC AGG TAC ATC TCG CCT GAC CAG CTG	GGG GTC ATC GGG AGC CTT GAA GTC CAG

**Sequence for siRNA**	
CD47-siRNA1	AAACGATCATCGAGCTAAA
CD47-siRNA2	GGCGTGTATACCAATGCAT
ENO1-siRNA1	AATGTCATCAAGGAGAAATAT
ENO1-siRNA2	CGTGACCGAGTCTCTTCAGGC

Supplementary Methods and Metarials

RNA extraction and qRT-PCR analysis

TRIzol Reagent (Invitrogen, CA, USA) was used according to the manufacturer's instructions to extract total RNA from cell lines and tissue specimens. The ReverTra Ace qPCR RT Kit (Toyobo, Japan) was used in reverse transcription reaction. ABI QuantStudio™ 7 Flex Real-Time PCR Systems (Applied Biosystems, USA) and SYBR Green PCR Master Mix (Applied Biosystems, USA) was used to perform the real-time PCR according to the manufacturer's protocols. The relative expression of target genes was normalized by β-actin using the 2-ΔΔCt method. The primer sequences for each gene are included in Supplementary Table S3.

Immunoblotting

Cell lysates for western blot were extracted from culture dishes in RIPA lysis buffer (150mM NaCl, 50mM Tris, 1% Nonidet P-40, 0.25% Na-deoxycholate, 1mM EDTA, pH 7.4) supplemented with a cocktail of protease/phosphatase inhibitors (Thermo Scientific, IL, USA). Lysates were sonicated for 10 minutes and then centrifugated at 4°C for 15minutes at 12,000g. Protein samples (30ug for each line) were separated by 10% sodium dodecyl sulphate-polyacrylamide gel electrophoresis (SDS-PAGE) and then transferred to polyvinylidene fluoride (PVDF) membranes (Millipore, MA, USA). The PVDF membranes were blocked with phosphate buffered saline (PBS) / Tween-20 containing 5% skim milk (BD Biosciences) for 1 hour at room temperature, and then probed with primary antibodies (Supplementary Table S1) overnight at 4°C. After the membranes were incubated with horseradish peroxidase (HRP) conjugated secondary antibodies (CST, USA) for 1 hour at room temperature, the ChemiDoc Touch Imaging System (Bio-Rad, USA) and Pierce™ ECL Western Blotting Substrate (Thermo, USA) were used to detect the specific bands.

Co-immunoprecipitation (co-IP), Immunopurification and mass spectrometry

HEK293T cells transfected with Flag-CD47 for 48h were lysed in Pierce IP lysis buffer containing protease inhibitors cocktail (Thermo Scientific, IL, USA) for 30 min at 4°C. Cell lysates were centrifugated at 13,000g for 20 min at 4°C, and protein supernatant was incubated with Mouse mAb IgG XP® Isotype Control (Magnetic Bead Conjugate, CST) and Anti-Flag Magnetic Beads (Bimake, MA, USA) overnight at 4°C. Magnetic beads-based immunoprecipitation was carried out according to the manufacturer's instruction. After washing with washing buffer (0.1% Triton-100X, 50 mM TrisCl, pH 7.4, 300 mM NaCl, 5 mM EDTA, 0.02% sodium azide) for five times, the beads were incubated with 1x sample loading buffer and boiled for 10 minutes. The lysate samples were then resolved on SDS-PAGE, Coomassie brilliant blue stained and processed for proteomic data analysis by LC-MS/MS at Jingjie PTM BioLab (Hangzhou, China). For co-IP assay, HEK293T cells co-transfected with indicated plasmids were lysed in Pierce IP lysis buffer containing protease inhibitors cocktail for 30 min at 4°C. Cell lysates were centrifugated at 13,000g for 20 min at 4°C, and 100 µL supernatant was used as input. The remaining supernatant was incubated with Mouse mAb IgG XP® Isotype Control, Anti-Flag Magnetic Beads, Anti-HA Magnetic Beads and Anti-Myc Magnetic Beads (Bimake, MA, USA) respectively overnight at 4°C. Magnetic beads-based immunoprecipitation was carried out according to the manufacturer's instruction. After

washing with washing buffer for five times, the beads were incubated with 1x sample loading buffer and boiled for 10 minutes. The co-IP and input samples were then detected by immunoblotting assays.

Ubiquitination and protein stability Assay

For ubiquitination assay, HEK293T cells were co-transfected with indicated siRNA and plasmids (siCD47, pSin-CD47, HA-ENO1 and Ubiquitin) for 48h, and cells were treated with 25μM MG132 for 6 hours before harvest. The cell lysates were prepared as described above in the co-IP part, and lysates were immunoprecipitated by the Anti-HA Magnetic Beads (Bimake, MA, USA). IP lysates and input samples were resolved by SDS-PAGE and incubated with specific antibodies. For protein stability assay, CRC cells were treated with 50μg/ml Cycloheximide (CHX, CST) for 0, 2, 4, 6, 8 hours before harvesting. And in some experiments, CRC cells were treated with MG132 (25μM, Calbiochem) for 12 h to inhibit proteasome activity. The cell lysates were harvest as described above and the protein levels were detected by immunoblotting.

Seahorse XF Extracellular Flux Analysis

The Seahorse Extracellular Flux Analyzer XF96 (Seahorse Bioscience, North Billerica, MA, USA) was used to measure the in vitro cells extracellular acidification rate (EACR) and oxygen consumption rate (OCR) based on the manufacturer's instructions. Briefly, 1×10^4 cells were seeded per well in the XF96-well cell culture plate and incubated at 37℃ overnight. Next day, medium was changed to bio-carbonate free DMEM with 1mM glutamine and then cells were incubated at 37℃ for 60 min in the CO_2 free incubator to balance the media pH and temperature. The ECAR and OCR were monitored in baseline conditions and treated with 10mM glucose, 1μM oligomycin, 50mM 2-deoxy glucose (2-DG), 1μM carbonyl cyanide 4-(trifluoromethoxy) phenylhydrazone (FCCP) or 0.5μM rotenone/antimycin. Data were normalized by the protein quantification.

Tissue Microarray and Immunohistochemistry

Paraffin-embedded CRC tissue blocks from the Sixth Affiliated Hospital of Sun Yat-sen University were used to construct tissue microarray slides. Standard immunohistochemistry stainings were performed as previously described [1]. In brief, the slides were put in a dry oven at 65°C for 30 minutes and then deparaffinized in xylene, rehydrated in graded ethanol. The slides were incubated in 0.3% H_2O_2 for 15 min to block endogenous peroxidase activity, and then processed for antigen retrieval by high pressure method for 25 minutes in 10 mM sodium citrate (pH 6.0). The slides were blocked using PBS containing 1% BSA for 60 min and subsequently incubated with specific primary antibody (Supplementary Table S1) overnight at 4°C. The slides were stained with diaminobenzidine (DAKO) and then counterstained with hematoxylin (Sigma, German). For quantification, the slides were assessed by two pathologists independently by using the Image Scope software. And the protein levels were evaluated based on the intensity and area of the staining. The intensity was scored by 3 groups (grade 0 with no staining, grade 1 with weak staining, grade 2 with medium staining and grade 3 with strong staining). The area of staining was determined by the percentage of positive stained area (grade 0=0, grade 1, 1-25%, grade 2, 26-50%, grade 3, 51-75% and grade 4>75%). To obtain the final

score, we multiplied the extent by grades of intensity staining. Then the samples were ranked by the final score. Ultimately, the protein expression was sorted into high expression and low expression by the median of the sample size. The X-tile and R (version 3.4.4) were used to optimize the cut-off point and conduct the survival analysis.

Plasmid construction and Cell Transduction

The specific primers used for plasmid construction were listed in supplementary table 3. The coding sequence (CDS) of CD47, ENO1 and FBXW7 were amplified using ClonExpress II One Step Cloning Kit (Vazyme, China), then the cDNA was inserted into the lentiviral expression vector pSin-EF2-Puro (transformed from pSin-EF2-Sox2-Puro, Addgene, USA). Short hairpin RNA (shRNA) targeting CD47 were cloned into pLKO.1-puro (Addgene, USA). Ubiquitin wild type plasmid is a gift from Dr. Wan of the Sixth Affiliated Hospital. The mutant HA-ENO1 (S263A/S268A) plasmid was constructed using KOD-Plus-Mutagenesis Kit (Toyobo, Japan) according to manufactor's instruction. HEK 293T cells were co-transfected with the pSin-CD47/ pLKO.1-shCD47, psPAX2 and pMD2.G (Addgene, USA) using Lipofectamine® 3000 (Invitrogen) to produce lentivirus. Six hours after transfection, the medium was replaced. And the supernatant was harvested at 24h, 48h, 72h post-transfection. CRC cells were infected with above supernatant in the presence of polybrene and selected with puromycin for 3 weeks after infection. The resistant colonies were selected and expanded for the sequent research under selective conditions.

RNA interference

Small interfering RNA (siRNA) for CD47 and ENO1 were purchased from RiboBio (Guangzhou, China). CRC cells were transfected with above siRNA using Lipofectamine® 3000 (Invitrogen). CRC cells were used for immunoblotting, RNA extraction, migration/invasion and proliferation assay after 48 hours of the transfection.

Real-Time Cell Proliferation Assay

The xCELLigence Real-Time Cell Analyzer (RTCA) system from ACEA Biosciences was used to conduct the real-time cell proliferation assays. Simply put, 50μL of cell culture media was pipetted to each well of the electronic plate to perform background corrections. And then we seeded 3×10^3 cells in 100μL media into the electronic plate and placed it on the RTCA instrument located in the 5% CO2-humidified incubator.

The Cell Index values were recorded automatically every 24 hours for 3 days.

Cell counting assay

SW480 and DLD1 cells were seeded in 6cm cell culture dish (Corning, USA) at the density of 2×10^5 cells and treated with 5μM ENOblock. These cells were trypsinized

at 0, 24, 48 and 72 hours after treatment. Then, cells were stained with trypan blue (Gibco, USA) and counted using Nexcelom Cellometer (Nexcelom, USA).

MTT Assay

MTT (Sigma, USA) assay was used to test the response of CRC cells treated by ENOblock. CRC cells were seeded in 96-well plates (Corning, USA) at the density of 4000 cells per well.

Then, cells were treated with different dose of ENOblock (Selleck, USA) for 72h. MTT reagent (20μl/well, Sigma, USA) was added to the plates and incubated for 2h. Finally, the microplate spectrophotometer (Thermo Scientific, USA) was used to to obtain the absorbance at 570nm.

Cell migration and invasion assays

We inserted 8-μm pore size chamber (Corning, USA) without Matrigel (for migration assays) or with Matrigel (for invasion assays) in a 24-well plate. CRC cells (5×10^4) were suspended in 200μL serum-free cell culture media and seeded on the upper chamber per well. And cell culture media containing 10% FBS was added to the lower chamber. After 48 hours of incubation at 37°C, the migrated cells were fixed 4% paraformaldehyde (Sangon, China) for 15 minutes, stained with crystal violet (Sangon, China) for 10 minutes. Then, the migrated cells were imaged and counted under microscopy (Olympus, Japan). The above experiments were conducted with three biological repeats and the average number of migrated cells per microscopic field in five fields per chamber were calculated.

Immunofluorescence

CRC cells (2×10^4) were plated on the coverslips (Corning, USA) and cells were fixed with 4% paraformaldehyde for 15 minutes at room temperature, permeabilized with 0.2% Triton X-100 (Sigma, Germany), blocked with 10% bovine serum albumin (BSA, Sigma, Germany) for 1 hour, and probed with specific primary antibody at 4°C overnight. The next day, the cells were incubated with Alexa Fluor 488 conjugated donkey anti-rabbit IgG and Alexa Fluor 594 conjugated donkey anti-mouse IgG (Invitrogen, USA) for 1 hour at room temperature. And then, the slides were mounted by Anti-fade Fluoresence Mounting Media with DAPI (Helixgen, China). The laser-scanning confocal microscope (TCS SP8 X, Leica, Germany) was used to capture the images.

***In Vivo* Experimentation**

Four-week-old BALB/C-nu/nu nude male mice were purchased and maintained in pathogen-free conditions at the Experimental Animal Center of Sun Yat-sen University. To establish the subcutaneous tumor model, 4×10^6 cells (SW480-shScramble, SW480-shCD47, DLD1-vector and DLD1-CD47) were injected subcutaneously into nude mice. To study function of CD47 on CRC *in vivo* with macrophage depletion, 3×10^6 cells (SW480-shScramble, SW480-shCD47) were implanted subcutaneously into the right flank of nude mice. The subcutaneous tumor diameters were measured with a caliper every 3 days and tumor volume was calculated using the formula: Volume= (Longer diameter × Shorter diameter^2)/2. Three weeks (eighteen days for macrophage depletion group) after injection, the tumors were excised, fixed with 10% formalin and embedded in paraffin for further analysis (H&E and IHC). To construct the pulmonary metastasis model, 4×10^6 cells (SW480-shScramble, SW480-shCD47, DLD1-vector and DLD1-CD47) were injected into the tail veins of nude mice. Two months after injection, the mice were sacrificed and the number of metastatic nodules on the lung surface were counted. The lung tissues were fixed with 10% formalin (Thermo, USA) and embedded in paraffin for further analysis (H&E and IHC). To study the potential therapeutic effects of ENO1 inhibitor (ENOblock) on CRC *in vivo*, SW480 cells (6×10^6) and DLD1 cells (6×10^6) were implanted

subcutaneously into the right flank of nude mice. Mice were divided into two groups according to their volumes a week following implantation. ENOblock (Selleck, USA) was injected intraperitoneally (10 mg/kg/day) every two days for two weeks. Tumor diameters were measured with caliper every 3 days and tumor volume was calculated using the formula: Volume= (Longer diameter × Shorter diameter^2)/2. Two weeks following treatment, the tumors were excised, fixed with 10% formalin and embedded in paraffin for further analysis (H&E and IHC). All animal experiments were conducted in accordance with the approval of the Institutional Animal Care and Use Committee of Sun Yat-sen University.

In vivo depletion of colonic macrophages

BALB/C-nu/nu nude male mice received intraperitoneal injection of liposome-encapsulated clodrolate (0.3 mL, Vrije Universiteit Amsterdam) or liposome-encapsulated PBS 2 days before cancer cell implantation, and on day 2, 6, 10, and 14 (see Supplementary Figure. 3A). Splenocytes isolated from the nude mice in each group were stained with CD11b and F4/80 antibodies and then subjected to flow cytometry analysis. The macrophage depletion efficiency was determined by the relative percentage of F4/80+/CD11b+ cells in the spleen.

Metabolite Extraction and Liquid Chromatography Tandem Mass Spectrometry (LC-MS/MS) analysis

For metabolite extraction, fresh media was added to cells three hours prior to harvesting. The media was then removed and the cells were washed with ice cold PBS twice before lysing. Cold 80% methanol/water (v/v, -80°C) was added to extract polar metabolites. The cell lysates were centrifuged at 15000 rpm for 15 min and the supernatant was dried with SpeedVac vacuum concentrators (Thermo Fisher Scientific, USA). The dried samples were subjected for LC-MS/MS analysis at Metabo-Profile Biotechnology (Shanghai, China). The metabolite peak areas were normalized by the respective protein concentrations.

Statistical Analysis

All data are shown as mean ± standard deviation (SD) from no less than three independent experiments. Log-rank tests and Kaplan-Meier methods were used to conduct survival analysis. The comparison between quantitative data with normality and homogeneity was analyzed by Student's t test or one-way analysis of variance (ANOVA). The $\chi 2$ test or Fisher Exact test was used to analyze qualitative variables of clinicopathological data. Wilcoxon tests were used to analyze CD47 and ENO1 RNA and protein levels between CRC and matched adjacent tissues. The correlation analysis was conducted using the Pearson's correlation test. Univariable and multivariable Cox proportional hazards regression were used to evaluate the prognostic factors in CRC patients. GraphPad Prism 6 was used to generate figures. SPSS 22.0 and R program (Version 3.4.4) was used to perform the statistical analysis. Statistical analysis was two-sided and p-values less than 0.05 were regarded as statistically significant.

[1] X.R. Wu, X.S. He, Y.F. Chen, R.X. Yuan, Y. Zeng, L. Lian, Y.F. Zou, N. Lan, X.J. Wu, P. Lan, High expression of CD73 as a poor prognostic biomarker in human colorectal cancer, J Surg Oncol, 106 (2012) 130-137.
